# *Drosophila* studies support a role for a presynaptic synaptotagmin mutation in a human congenital myasthenic syndrome

**DOI:** 10.1371/journal.pone.0184817

**Published:** 2017-09-27

**Authors:** Mallory C. Shields, Matthew R. Bowers, McKenzie M. Fulcer, Madelyn K. Bollig, Patrick J. Rock, Bryan R. Sutton, Alysia D. Vrailas-Mortimer, Hanns Lochmüller, Roger G. Whittaker, Rita Horvath, Noreen E. Reist

**Affiliations:** 1 Department of Biomedical Sciences, Colorado State University, Fort Collins, CO, United States of America; 2 Molecular, Cellular, and Integrative Neuroscience Program, Colorado State University, Fort Collins, CO, United States of America; 3 School of Medicine, Texas Tech University Health Sciences Center, Lubbock, Texas, United States of America; 4 Department of Cell Physiology and Molecular Biophysics, Center for Membrane Protein Research, Texas Tech University Health Sciences Center, Lubbock, Texas, United States of America; 5 Department of Biological Sciences, University of Denver, Denver, CO, United States of America; 6 School of Biological Sciences, Illinois State University, Normal, IL, United States of America; 7 John Walton Muscular Dystrophy Research Centre, Institute of Genetic Medicine, Newcastle University, Newcastle upon Tyne, NE1 3BZ, United Kingdom; 8 Institute of Neuroscience, Newcastle University, Newcastle upon Tyne, NE2 4HH, United Kingdom; EPFL, SWITZERLAND

## Abstract

During chemical transmission, the function of synaptic proteins must be coordinated to efficiently release neurotransmitter. Synaptotagmin 2, the Ca^2+^ sensor for fast, synchronized neurotransmitter release at the human neuromuscular junction, has recently been implicated in a dominantly inherited congenital myasthenic syndrome associated with a non-progressive motor neuropathy. In one family, a proline residue within the C_2_B Ca^2+^-binding pocket of synaptotagmin is replaced by a leucine. The functional significance of this residue has not been investigated previously. Here we show that *in silico* modeling predicts disruption of the C_2_B Ca^2+^-binding pocket, and we examine the *in vivo* effects of the homologous mutation in *Drosophila*. When expressed in the absence of native synaptotagmin, this mutation is lethal, demonstrating for the first time that this residue plays a critical role in synaptotagmin function. To achieve expression similar to human patients, the mutation is expressed in flies carrying one copy of the wild type *synaptotagmin* gene. We now show that *Drosophila* carrying this mutation developed neurological and behavioral manifestations similar to those of human patients and provide insight into the mechanisms underlying these deficits. Our *Drosophila* studies support a role for this synaptotagmin point mutation in disease etiology.

## Introduction

Understanding the mechanisms mediating information transmission across a chemical synapse is essential to understanding brain function. During synaptic transmission, membranous vesicles filled with neurotransmitter must fuse with the presynaptic membrane in response to Ca^2+^ influx, thereby releasing the transmitter into the synaptic cleft. The synaptic vesicle protein, synaptotagmin, is essential for this release [1–3] as it binds the Ca^2+^ that triggers vesicle fusion [4–6]. Synaptotagmin has two Ca^2+^-binding pockets, C_2_A and C_2_B [7–9], which have both been extensively studied for their role in synchronizing vesicle fusion *in vivo* [4, 6, 10–12] and in culture [13, 14]. Synaptotagmin 2 is the predominant isoform found at mammalian neuromuscular junctions [15, 16], and until recently, has never been linked to disease. However, whole-exome sequencing techniques have now identified point mutations in the human *synaptotagmin 2 (syt2)* gene in patients with a disease combining congenital myasthenic syndrome and distal motor weakness, suggesting a role for synaptotagmin in neuromuscular disease [17, 18].

Two independent mutations in the C_2_B Ca^2+^-binding pocket of *syt2* have been identified: one in a United States (US) family and one in a United Kingdom (UK) family. Patients in each of the two families exhibit a novel neuromuscular syndrome characterized by foot deformities, fatigable ocular movements, lower limb weakness, and potentiation of tendon reflexes following exercise [17, 18]. In nerve conduction studies, all patients exhibited normal sensory responses. However, motor responses revealed low amplitude compound muscle action potentials (CMAPs) that exhibited significantly less amplitude depression following brief maximum voluntary contraction [17, 18]. These findings are indicative of a presynaptic deficit of neuromuscular junction function.

**Fig 1:**
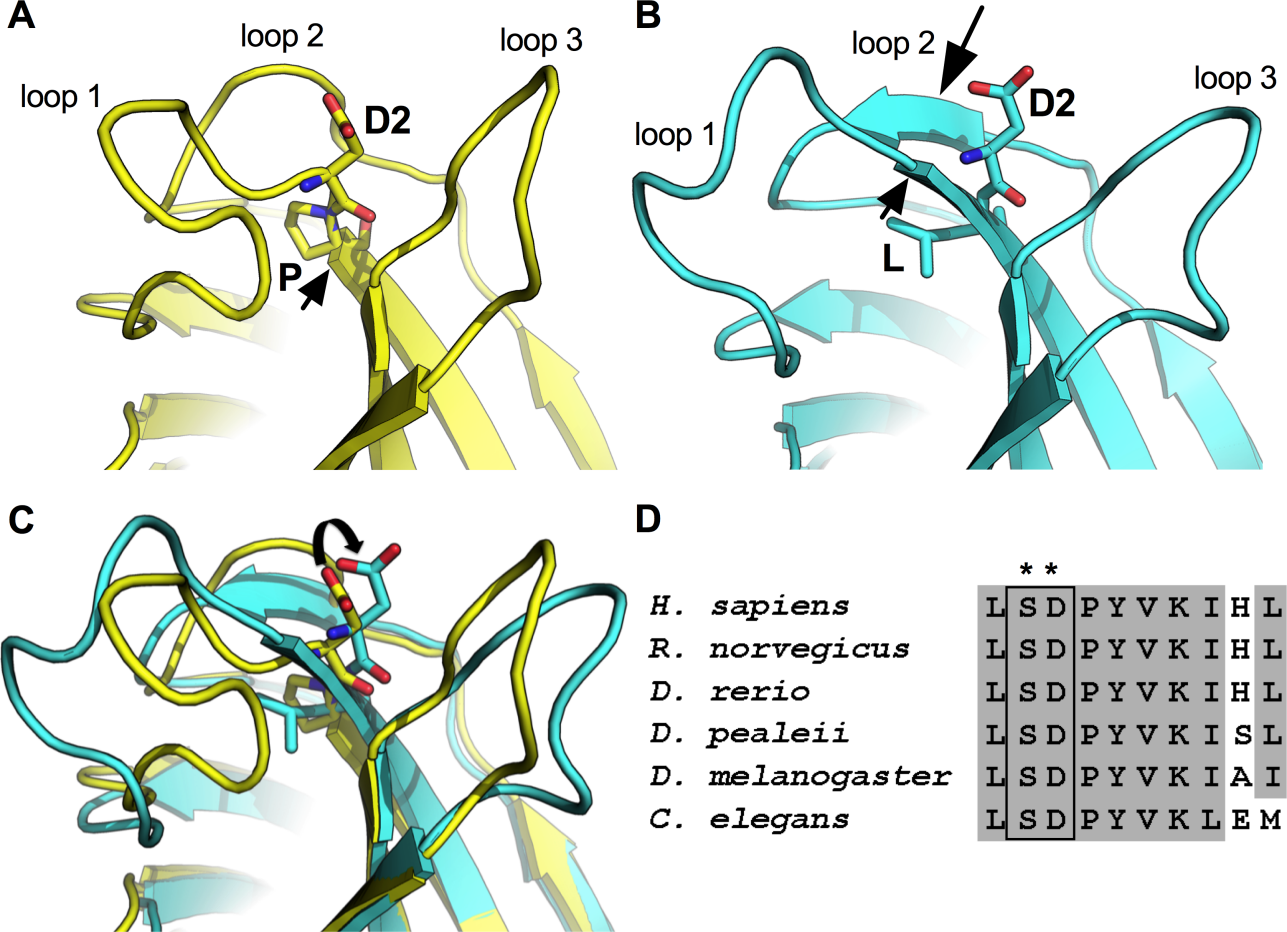
Predicted conformational changes in synaptotagmin’s C_2_B Ca^2+^-binding pocket. (A) Molecular model depicting the crystal structure of the synaptotagmin C_2_B Ca^2+^-binding pocket. Flexible loops 1, 2, and 3 are indicated. The proline residue under investigation (P) and the adjacent, Ca^2+^-coordinating aspartate (D2) are shown as stick models. Note that the proline is at one end of β-strand 3 (small arrow) and the aspartate is in loop 1. (B) Predicted structural changes induced by the syt^P-L^ substitution. The leucine (L) and the adjacent aspartate (D2) residues are shown as stick models and both are now within β-strand 3 (small arrow indicates extension of β-strand 3). The large arrow indicates a newly-formed β-strand. (C) Overlay of wild type (yellow) and syt^P-L^ mutant (turquoise). The curved arrow indicates the displacement of the D2 Ca^2+^-binding residue. (D) Sequence alignment of the highly conserved SDPYVK amino acid motif of the synaptotagmin C_2_B domain from humans, rat, zebra fish, squid, *Drosophila*, and *C*. *elegans*. * locations of the point mutations in the US and UK families, respectively. Note: *syt1* and *syt2* in mammals are identical in this region.

Interestingly, both familial mutations are located in C_2_B Ca^2+^-binding pocket of *syt2* within the highly conserved “SDPYVK” residue motif [Fig 1, [17, 18]]. The US family possesses an aspartate 307 (referred to as D2) to alanine substitution within this motif (Fig 1D, first *). As D2 is one of the well-ordered C_2_B aspartate residues known to coordinate the binding of Ca^2+^ [9, 19, 20] required for triggering neurotransmitter release [4, 11, 14], the function of this residue has been extensively studied. The UK family possesses a proline 308 to leucine substitution within this motif (Fig 1D, second *). We investigated the anticipated conformational changes of the proline to leucine (P-L) substitution by modeling this P-L mutation *in silico*. Simulation of the P-L mutation predicts that replacement of the highly conserved proline residue by a leucine would extend β-strand 3 into the Ca^2+^-binding pocket (Fig 1A and 1B small arrow). The extension of β-strand 3 is accompanied by a significant distortion of the backbone angles of residues on the amino terminal side of the proline (S1 Fig). The distortion of upstream residues places this critical D2 Ca^2+^-binding residue in a non-ideal conformation for Ca^2+^ binding (Fig 1C curved arrow, S1 Fig). Further, the additional amide proton from the mutant leucine residue forms a new H-bond with neighboring residues in loop 2 (not shown), thereby altering the shape, and likely the flexibility, of the Ca^2+^-binding pocket. A more rigid Ca^2+^-binding pocket, as well as the sub-optimally positioned D2 Ca^2+^-binding residue, predict deficiencies in Ca^2+^ binding in the P-L mutation. Together, our modeling predicts that this mutation would result in conformational changes in synaptotagmin’s C_2_B domain that would impair Ca^2+^ binding. Although this proline residue is highly conserved (Fig 1D), its importance for synaptotagmin function has not been investigated previously.

To directly assess whether the syt^P-L^ point mutation impacts Ca^2+^-binding resulting in synaptic deficits *in vivo*, we generated a homologous P-L mutation in the synaptotagmin isoform expressed at the *Drosophila* neuromuscular junction (*syt^P-L^*). In mammals, the *synaptotagmin 1* gene (*syt1*) is predominantly expressed throughout the cerebral hemispheres while *syt2* is predominantly expressed in the brainstem and spinal cord, and hence at the neuromuscular junction [15, 16]. There is no *syt2* gene in *Drosophila* [21, 22]; the *syt1* gene is expressed pan-neuronally [23, 24] and codes for the Ca^2+^ sensor for fast, synchronous neurotransmitter release [4, 5] throughout the nervous system. When the *syt^P-L^* mutation is expressed in *Drosophila* in the absence of wild type synaptotagmin, it results in lethality, demonstrating that this proline residue is critical for synaptotagmin function. As the human condition presents as a *syt* heterozygous dominant disorder, with patients carrying one copy of the wild type *syt* gene and one copy of the mutated *syt* gene (*syt^WT^/syt^P-L^*), we simulated this expression pattern by driving pan-neuronal expression from one mutant transgene using *elavGal4* in *syt* heterozygotes *(+/-;P[syt^P-L^]/+*). Physiologically, mutant larvae exhibit decreased evoked transmitter release and less synaptic depression during high-frequency stimulus trains, consistent with the human electrophysiological deficits. In addition, the decreased Ca^2+^ affinity of release and the associated decreased release probability documented here are consistent with the predicted disruption to the C_2_B Ca^2+^-binding pocket (Fig 1A–1C). Behaviorally, mutant adult flies exhibit less overall activity and a marked increase in fatigability, also consistent with the human phenotype. Together, these results indicate that this proline to leucine substitution impairs Ca^2+^ binding and support a role for this substitution in the etiology of this human neuromuscular disease.

## Results

### In the absence of wild type synaptotagmin, the *P[syt*^*P-L*^*]* mutation increases the lethality rate

To assess the effects of the *syt^P-L^* mutation in the absence of the wild type protein, we drove pan-neuronal expression of *syt* transgenes in the *syt^null^* background using the *elavGal4* promoter (referred to as *-/-;P[syt^WT^]/+* or *-/-;P[syt^P-L^]/+*, see methods for full genotype). Rates of survival to the first instar larval stage were also compared to larvae lacking any synaptotagmin 1 expression (*-/-*). Balancer chromosomes used in our crossing scheme result in embryonic lethality for any wild type synaptotagmin homozygotes (*+/+* for *syt*, included in Table 1 under unhatched) due to the presence of a homozygous lethal *Cy* mutation (see methods and S2 Fig) with an expected frequency of ~25%.

**Table 1:** The *syt^P-L^* mutation negatively impacts survival rate in the absence of native synaptotagmin.

	*P[syt^WT^]*	*P[syt^P-L^]*	No transgenic *syt*
***-/-***	25.7% (365)	12.0% (212)	18.0% (231)
***+/-***	48.7% (691)	50.1% (888)	49.8% (640)
**Unhatched**	25.6% (363)	37.9% (671)	32.2% (415)

Table 1 depicts percentages and raw counts (in parentheses) of first instar larvae in the *syt^null^* (*-/-*, top row) and *syt* heterozygous (*+/-*, second row) backgrounds and embryonic lethality (unhatched) of progeny containing one copy of synaptotagmin transgenes (*P[syt^WT^*] or *P[syt^P-L^]*) or of progeny containing no transgenic *syt* (last column). All crosses were carried out using the *CyO* second chromosome balancer such that *syt* homozygotes are lethal (included in unhatched) due to the presence of the homozygous lethal *Cy* mutation (*syt^WT^ Cy/syt^WT^ Cy*). There was a significant decrease in the percentage of first instars expressing one copy of *P[syt^P-L^]* in the absence of any wild type synaptotagmin (*-/-;P[syt^P-L^]/+*, 12.0%) compared to control (*-/-;P[syt^WT^]/+*, 25.7%, p < 0.0001, Chi-squared test). There was also a significant decrease in the percentage of first instars expressing one copy of the *P[syt^P-L^]* transgene (*-/-;P[syt^P-L^]/+*, 12.0%) compared to no synaptotagmin expression at all (*-/-*; no transgenic *syt*, 18.0%, p < 0.0001, Chi-squared test).

As expected for a fully functional protein, the wild type transgenic cross resulted in progeny where ~25% were *syt^null^* mutants expressing synaptotagmin from the wild type transgene (*-/-;P[syt^WT^]/+*; Table 1, 25.7%). This is significantly larger than in the *P[syt^P-L^]* cross, in which only 12.0% of progeny were *syt^null^* mutants expressing the mutated transgene (*-/-;P[syt^P-L^]/+*, Table 1, 12.0%, p < 0.0001). As previously shown, *syt^null^* mutants are viable [25], and the cross lacking any source of synaptotagmin resulted in 18.0% of progeny that were *syt^null^* mutants (Table 1, no transgenic *syt*, 18.0%). Yet the expression of the mutant transgene resulted in a significant decrease in the rate of survival to the 1^st^ instar larval stage (Table 1, 18.0% down to 12.0%, p < 0.0001). Thus, in the absence of wild type synaptotagmin, the expression of *P[syt^P-L^]* not only fails to rescue synaptotagmin function, it results in a negative impact on survival. This is the first study to show the critical nature of this proline residue for synaptotagmin function.

### Transgenic synaptotagmin is expressed appropriately

**Fig 2:**
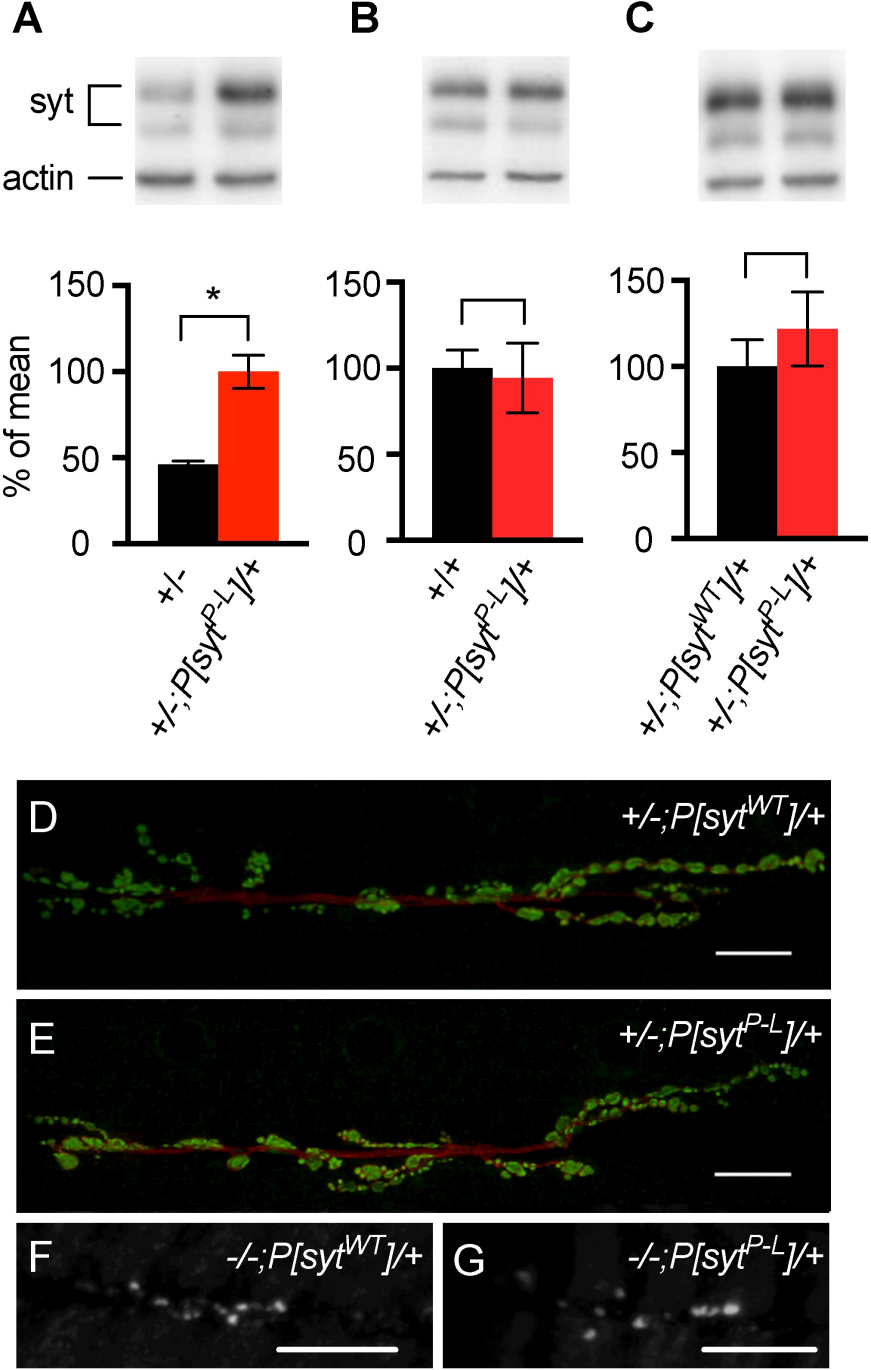
Transgenic synaptotagmin is expressed at appropriate levels and exhibits appropriate localization. (A-C) Representative western blots above and average synaptotagmin expression normalized to actin levels below. *Syt* heterozygotes expressing one copy of the mutant transgene (*+/-;P[syt^P-L^]/+*, n = 6, 5, and 7, respectively) are compared to *syt* heterozygotes (*+/-*, n = 5, A), *syt* homozygotes (*+/+*, n = 5, B), and *syt* heterozygotes expressing one copy of the wild type transgene (*+/-; P[syt^WT^]/+*, n = 8, C). The *P[syt^P-L^]* heterozygotes had approximately twice as much synaptotagmin expression as *syt* heterozygotes (p = 0.0008, student t-test), while there was no significant difference in expression between the other lines compared (Fig 2B, p = 0.69 and Fig 2C, p = 0.25). Error bars depict SEM. (D, E) Third instar larvae stained with antibodies against horseradish peroxidase (HRP) as a general axonal stain (red) and synaptotagmin (green). Scale bars represent 10 μm. (F, G) First instar larvae stained with antibodies against synaptotagmin. Scale bars represent 20 μm.

To verify appropriate transgenic synaptotagmin expression levels for modeling this disease in *Drosophila*, western blot analysis was performed on the central nervous system (CNS) of larvae. Comparison of CNS samples from *syt* heterozygotes (*+/-*) and *syt* heterozygotes expressing one copy of the mutant transgene (*+/-; P[syt^P-L^]/+*, hereafter referred to as *P[syt^P-L^]* heterozygotes) showed significant differences in the level of synaptotagmin expression with an approximate doubling of synaptotagmin levels in the transgenic line (Fig 2A, *+/-* is 46 ± 18% of *+/-;P[syt^P-L^]/+*, p = 0.0008). This finding indicates approximately equal protein expression from the native gene and the transgene. Consistent with this interpretation, CNS samples from wild type *syt* homozygotes (*+/+*) and from *P[syt^P-L^]* heterozygotes showed similar levels of synaptotagmin expression (Fig 2B, *+/-;P[syt^P-L^]/+* is 94 ± 9.0% of *+/+*, p = 0.69). Fig 2C demonstrates approximately equal levels of synaptotagmin in *P[syt^P-L^]* heterozygotes and the *P[syt^WT^]* heterozygous controls (*+/-; P[syt^P-L^]/+* is 121 ± 8.1% of *+/-;P[syt^WT^]/+*, p = 0.25). Thus, our heterozygous transgenic control also expresses appropriate levels of synaptotagmin. Finally, both the transgenic wild type and transgenic mutant protein are appropriately targeted to the synapse (Fig 2D–2G). As seen in wild type *Drosophila* [23, 24], synaptotagmin staining in muscles is restricted to the neuromuscular junction in third instar larvae of both the *P[syt^WT^]* heterozygote controls and the *P[syt^P-L^]* heterozygotes (Fig 2D and 2E, respectively). Staining of first instar larvae expressing the *syt* transgenes in the absence of native synaptotagmin also revealed that the transgene is appropriately expressed at the neuromuscular junction (Fig 2F and 2G, *-/-;P[syt^WT^]/+* and *-/-; P[syt^P-L^]/+*).

### Lifespan is decreased in *P[syt^P-L^]* heterozygotes

**Fig 3:**
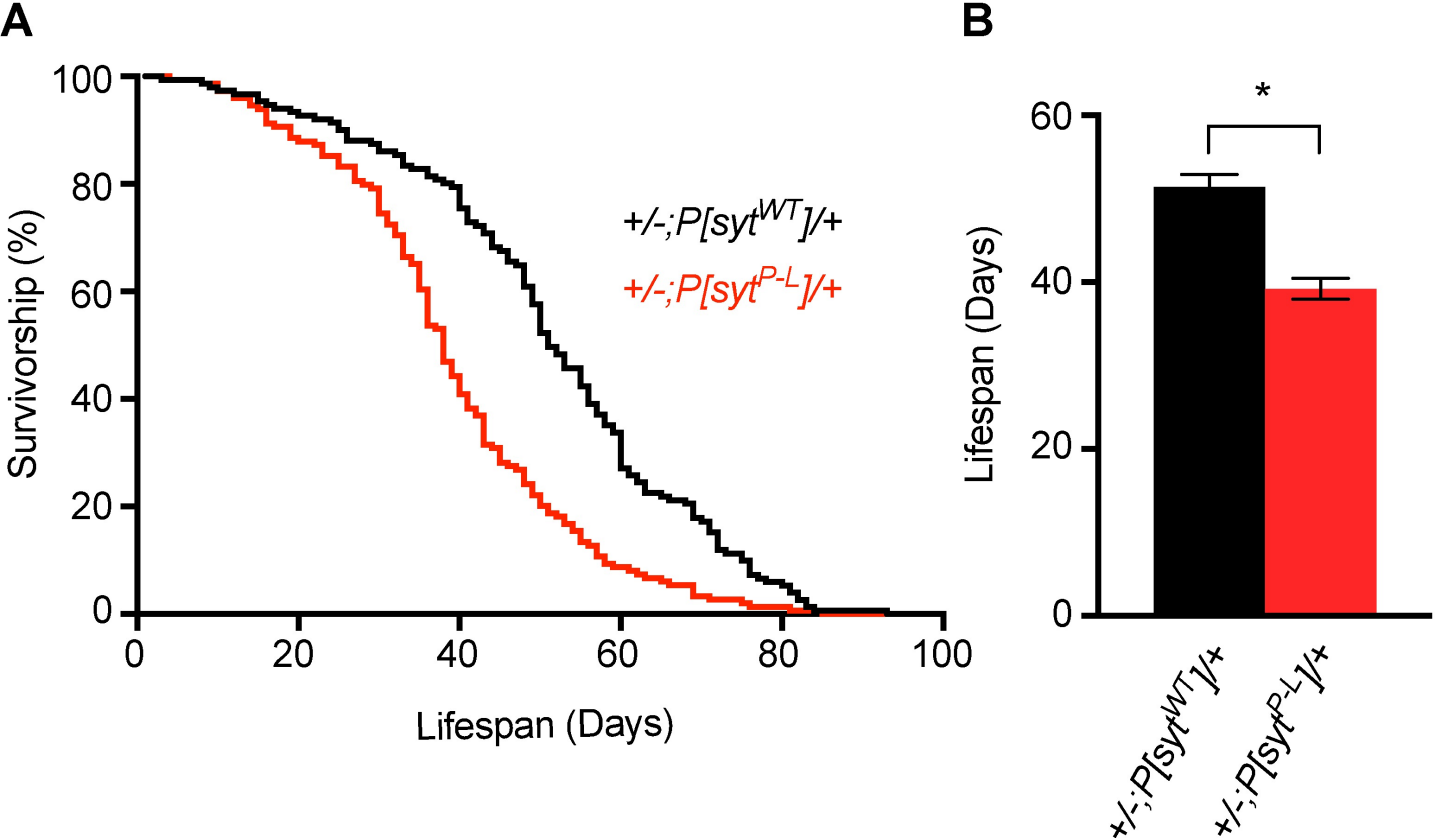
Lifespan is decreased in *P[syt^P-L^]* heterozygotes. (A) Adult lifespan curves of *+/-;P[syt^WT^]/+* (n = 151, black) and *+/-;P[syt^P-L^]/+* (n = 149, red). (B) Mean lifespan is significantly decreased in *+/-;P[syt^P-L^]/+* adults (p < 0.0001, Wilcoxon Rank-Sum test).

While *P[syt^P-L^]* heterozygotes are easily generated and maintained throughout all larval and adult stages, we wanted to ascertain whether this mutation had any impact on lifespan in heterozygotes. Therefore, we performed a longevity assay. Lifespans between *P[syt^WT^]* heterozygotes (51.4 ± 1.50 days, mean lifespan ± SEM, n = 151) and *P[syt^P-L^]* heterozygotes (39.2 ± 1.25 days, mean lifespan ± SEM, n = 149) are significantly different (Fig 3, p < 0.0001, Wilcoxon Rank-Sum test). However, this does not relate directly to the human condition, who show no indication of this P-L mutation impacting longevity, as these flies do not receive any medical intervention. These results support a detrimental role of the proline to leucine mutation in survival.

### The *syt^P-L^* mutation disrupts evoked transmitter release

**Fig 4:**
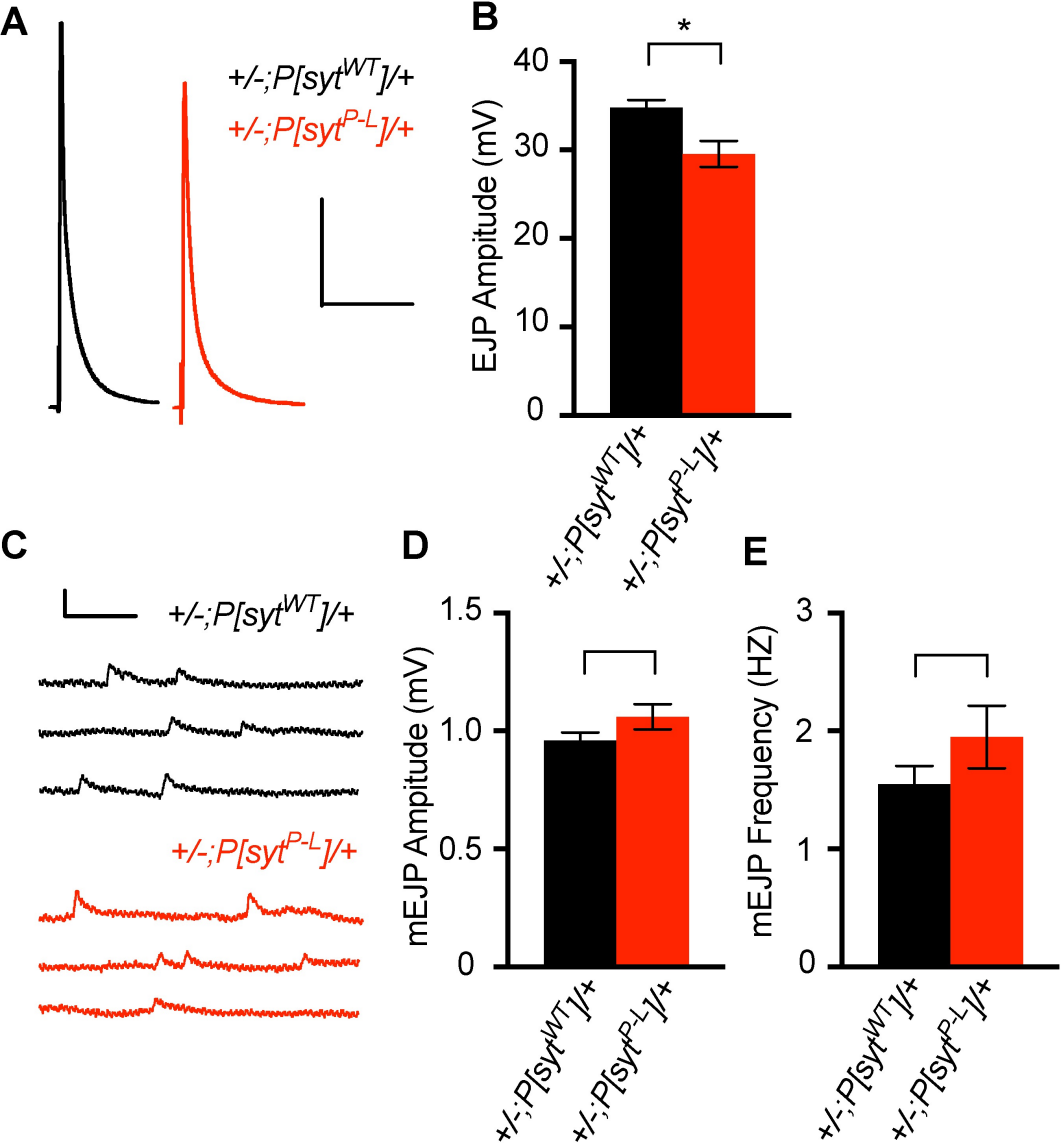
Synchronous evoked release is impaired in *P[syt^P-L^]* heterozygotes, but quantal content is unchanged. (A) Representative EJP traces for control and *P[syt^P-L^]* heterozygotes. Scale bars represent 10 mV, 0.2 s. (B) Mean EJP amplitude is significantly less in *P[syt^P-L^]* heterozygotes (n = 19) compared to control (n = 12, *p = 0.002). Error bars depict SEM. (C) Representative consecutive 3 second mEJP traces for control and *P[syt^P-L^]* heterozygotes. Scale bars represent 1 mV, 0.2 s. (D, E) Neither mean mEJP amplitude (p = 0.09, Wilcoxon Rank-Sum Test, D) nor mean mEJP frequency (p = 0.18, student t-test, E) is significantly different between *P[syt^P-L^]* heterozygotes (n = 12 fibers) and control (n = 19 fibers). Error bars depict SEM.

In human motor nerve conduction studies, the summated response of all muscle fibers supplied by a particular motor nerve can be measured as a CMAP. The *syt^P-L^*-affected family members present with decreased CMAP amplitudes [17, 18], consistent with a decrease in evoked transmitter release. To test evoked release in *Drosophila P[syt^P-L^]* heterozygotes, we measured excitatory junction potentials (EJPs) at the larval neuromuscular junction. Upon nerve stimulation, *P[syt^P-L^]* heterozygotes exhibited a significant decrease in the amount of neurotransmitter released (Fig 4A and 4B). Average EJP ± SEM for *+/-;P[syt^WT^]/+* was 34.8 ± 0.83 mV, and for *+/-;P[syt^P-L^]/+* was 29.6 ± 1.5 mV (p = 0.002, student t-test). This decrease in release is consistent with the decrease in baseline amplitude in the human motor nerve conduction studies.

One potential explanation for this observed 20% decrease in evoked response could be a decrease in quantal content. To determine quantal size, we calculated the mean amplitudes of Ca^2+^-independent spontaneous miniature EJPs (mEJPs) in both *P[syt^P-L^]* heterozygotes and their controls. Each mEJP results from one vesicle fusing with the presynaptic membrane. Thus, the amplitude of the response is an indication of the amount of neurotransmitter loaded into each vesicle. Neither the mean amplitude nor frequency of mEJPs in controls and *P[syt^P-L^]* heterozygotes were significantly different (Fig 4C–4E). Mean mEJP amplitude ± SEM for *P[syt^WT^]* heterozygotes was 0.96 ± 0.03 mV (n = 50 mEJP events from 19 fibers), and for *P[syt^P-L^]* heterozygotes was 1.06 ± 0.05 mV (n = 50 mEJP events from 12 fibers, p = 0.09, Wilcoxon Rank-Sum Test). Mean mEJP frequency ± SEM for *P[syt^WT^]* heterozygotes was 1.55 ± 0.15 Hz (n = 19 fibers), and for *P[syt^P-L^]* heterozygotes was 1.94 ± 0.26 Hz (n = 12 fibers, p = 0.18, student t-test). These results demonstrate that the decrease in evoked release is not due to a change in quantal content.

**Fig 5:**
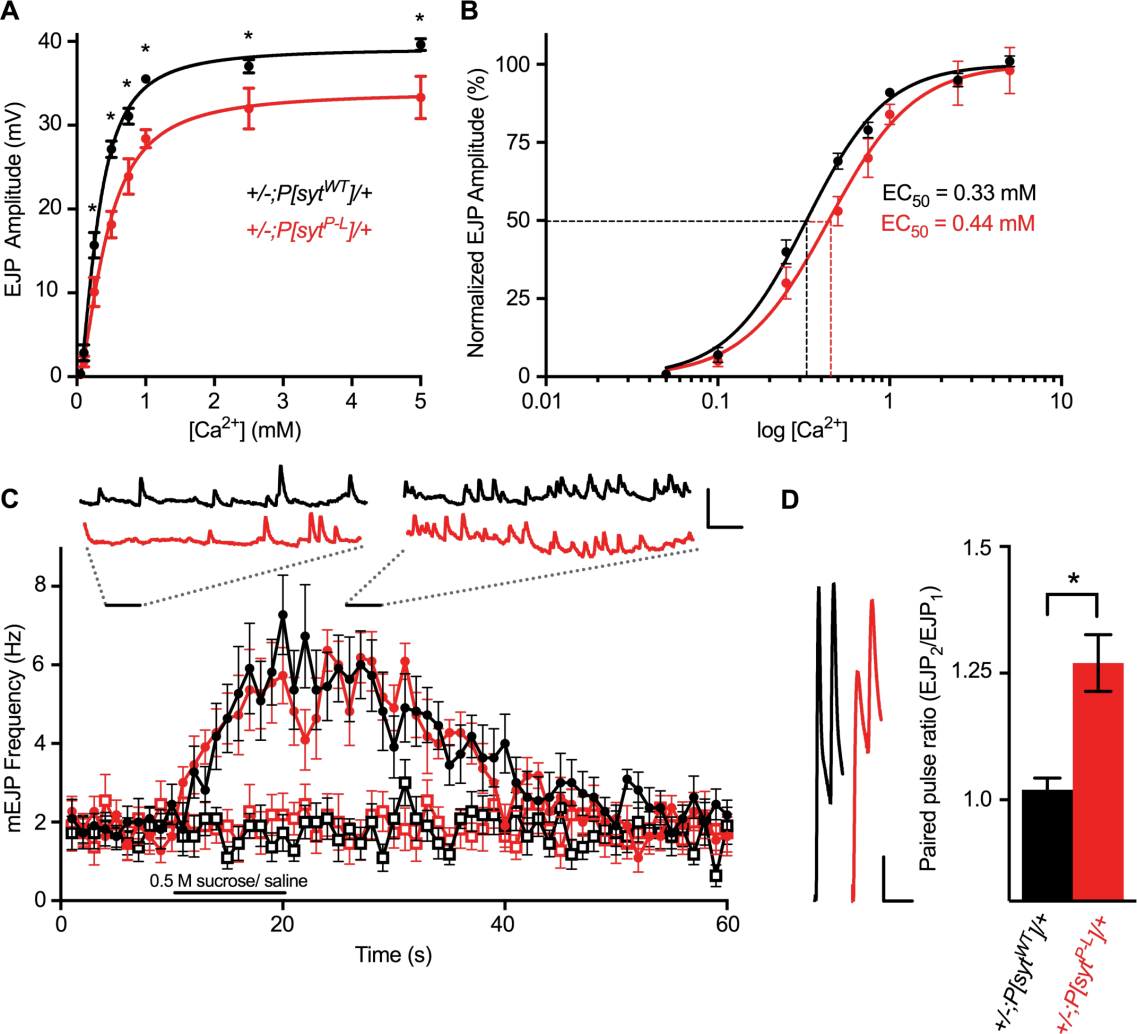
The *P[syt^P-L^]* mutation decreases the Ca^2+^ affinity of release and vesicular release probability, but the readily releasable pool is unchanged. (A) Mean EJP amplitudes from *+/-;P[syt^WT^]/+* (filled black circles) and *+/-;P[syt^P-L^]/+* (filled red circles) larvae at Ca^2+^ levels ranging from 0.05 mM—5.0 mM. At Ca^2+^ concentrations above 0.1 mM, statistical differences are seen between the two genotypes (*p < 0.05 for indicated Ca^2+^ concentrations, see S1 Table for specific n’s and p-values, student t-tests). Lines of best fit were determined using non-linear regression analyses. (B) Graph depicting normalized EJP responses at log Ca^2+^ concentrations. EC_50_ values were determined using the line of best fit determined by nonlinear regression analyses. Dotted lines depict the shift in the EC_50_ value. (C) Application of hypertonic 0.5 M sucrose to *+/-;P[syt^WT^]/+* (n = 11 fibers, filled black circles) and *+/-;P[syt^P-L^]/+* (n = 11 fibers, filled red circles) results in increases in mEJP frequency. Black bar below the traces represents the duration of the 10 second application of 0.5 M sucrose or saline solution. Black bars above the mEJP responses indicate three consecutive seconds of individual traces either before sucrose application (left inset) or during peak sucrose response (right inset). Similar frequencies between *+/-;P[syt^WT^]/+* and *+/-;P[syt^P-L^]/+* are observed both before and during sucrose response (n = 11 fibers for both genotypes, p = 0.28 before sucrose application, p = 0.23 during maximum sucrose response). Application of bath solution does not result in an increase in mEJP frequency in either genotype (open circles) (p = 0.57 for *+/-; P[syt^WT^]/+* and p = 0.83 for *+/-;P[syt^P-L^]/+*, student t-test). Scale bar 2mV, 3s. (D) Paired-pulse analysis indicates a decrease in release probability in *+/-;P[syt^P-L^]/+*. Left, representative traces of responses stimulated with a 20 ms interpulse interval. Scale bar 5 mV, 40 ms. Right, in *P[syt^P-L^]* heterozygotes, the paired pulse ratio is significantly increased (*+/-;P[syt^WT^]/+* = 1.02 ± 0.02, *+/-;P[syt^P-L^]/+* = 1.27 ± 0.06, *p = 0.0008, student t-test). Error bars are SEM.

Transmitter release from an activated nerve terminal is a Ca^2+^-dependent, cooperative process [26]. Evoked release was measured at a variety of extracellular Ca^2+^ concentrations, ranging from 0.05 mM to 5 mM, to assess whether the Ca^2+^ dependence of release was altered in *P[syt^P-L^]* heterozygotes. At all Ca^2+^ concentrations above 0.1 mM, *P[syt^P-L^]* heterozygotes exhibit a decrease in evoked release compared to controls (Fig 5A). A nonlinear regression analysis determined a change in the Ca^2+^ dependence of *P[syt^P-L^]* heterozygotes. The extracellular Ca^2+^ concentration at which a 50% maximum response is reached (EC_50_) is statistically shifted in *P[syt^P-L^]* heterozygotes compared to controls. In *+/-;P[syt^WT^]/+* EC_50_ = 0.33 mM Ca^2+^ (95% confidence intervals from 0.30–0.36 mM Ca^2+^) and in *+/-;P[syt^P-L^]/+* EC_50_ = 0.44 mM Ca^2+^ (95% confidence intervals from 0.41–0.48 mM Ca^2+^, Fig 5B). The non-overlapping confidence intervals demonstrate that the proline to leucine substitution in synaptotagmin results in a significant decrease in the Ca^2+^ affinity of release.

### Release probability is decreased in *P[syt^P-L^]* heterozygotes

A decrease in the size of the readily releasable pool and/or a decrease in release probability for individual vesicles could result in decreased evoked transmitter release. To determine whether *P[syt^P-L^]* heterozygotes exhibit alterations in the size of their readily releasable pool of synaptic vesicles, we triggered Ca^2+^-independent vesicle fusion events using a hypertonic solution (0.5 M sucrose). Before sucrose stimulation, mEJP frequency is similar between both *P[syt^WT^]* and *P[syt^P-L^]* heterozygotes [Fig 5C filled symbols, *+/-;P[syt^WT^]/+* (black), 1.95 ± 0.07 Hz and *+/-;P[syt^P-L^]/+* (red), 1.81 ± 0.10 Hz, mean mEJP frequency ± SEM, n = 11 fibers for both genotypes, p = 0.28, student t-test). Increases in mEJP frequencies are observed in both controls (p < 0.0001, student t-test) and *P[syt^P-L^]* heterozygotes (p < 0.0001, student t-test) upon sucrose stimulation. These mEJP frequencies are also similar between genotypes during the maximum sucrose response period (Fig 5C filled symbols, *+/-;P[syt^WT^]/+*, 5.62 ± 0.23 Hz and *+/-;P[syt^P-L^]/+*, 5.32 ± 0.23 Hz, mean mEJP frequency ± SEM, p = 0.23, student t-test). When HL3.1 solution is puff applied, mEJP frequency remains similar between *P[syt^WT^]* and *P[syt^P-L^]* heterozygotes (Fig 5C open symbols, *+/-;P[syt^WT^]/+*, 1.71 ±.0.10 Hz and *+/-;P[syt^P-L^]/+* = 1.96 ± 0.10 Hz, mean mEJP frequency ± SEM, p = 0.23, student t-test, n = 11 for both genotypes). Moreover, no increase is observed for either genotype during or after saline stimulation (Fig 5C, open circles, *+/-;P[syt^WT^]/+* p = 0.57 and *+/-;P[syt^P-L^]/+* p = 0.83, student t-test), indicating the increases in mEJP frequencies observed during sucrose stimulation result from hypertonic solution directly, and not a mechanical stimulation due to the puff application. Thus, the size of the readily releasable pool is not significantly altered in *P[syt^P-L^]* heterozygotes.

To address release probability, we used a paired-pulse analysis examining the ratio of the second response divided by the first response using a 20 msec interpulse interval. *P[syt^WT^]* heterozygotes (n = 18) and *P[syt^P-L^]* heterozygotes (n = 24) result in significantly different paired pulse ratios [Fig 5D, *+/-;P[syt^WT^]/+* = 1.02 ± 0.02 (first pulse = 34.4 ± 0.84 mV, second pulse = 35.2 ± 0.89 mV, EJP amplitude ± SEM), compared to *+/-;P[syt^P-L^]/+* = 1.27 ± 0.06 (first pulse = 25.1 ± 1.02 mV, second pulse = 30.6 ± 0.84 mV, EJP amplitude ± SEM), p = 0.0008, student t-test]. The significantly larger paired pulse ratio indicates a decrease in release probability in *P[syt^P-L^]* heterozygotes. Thus, our electrophysiological analysis of *P[syt^P-L^]* heterozygotes indicates the decrease in evoked transmitter release is not due to changes in the readily releasable pool, but rather results from a decrease in vesicular release probability.

**Fig 6:**
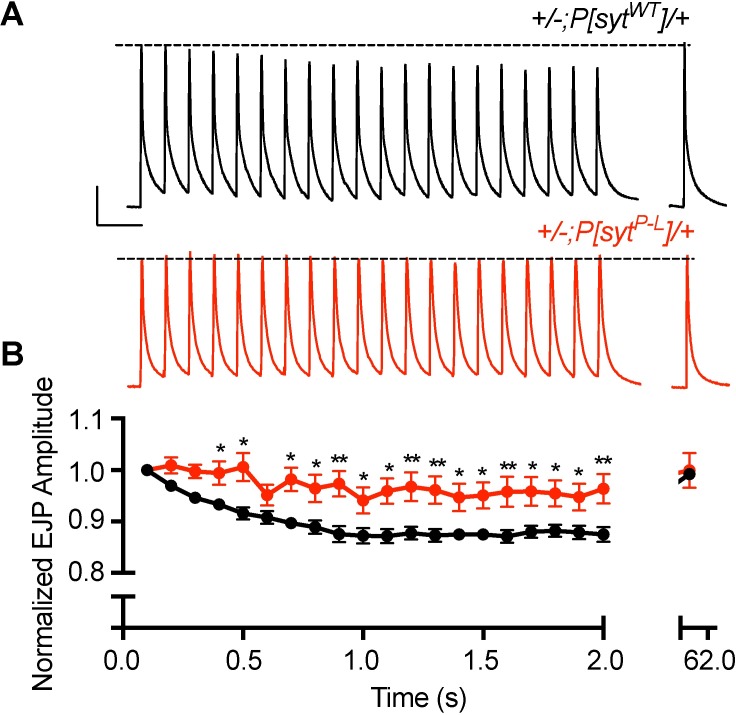
*P[syt^P-L^]* heterozygotes exhibit less depression throughout the course of high frequency stimulation, but fail to maintain this relatively increased response upon cessation of stimulation. (A) Representative traces from control and *P[syt^P-L^]* heterozygotes during a 2 second, 10 Hz stimulation train, and a single stimulus 1 minute following cessation of the train. Dotted line represents the amplitude of the initial response. Scale bar represents 10 mV, 0.2 s. (B) Mean EJP amplitudes of *P[syt^P-L^]* heterozygotes (n = 17) showed less depression compared to control (n = 14, *indicates p < 0.05, and **indicates p < 0.001). However, this relative increase in release is not maintained upon cessation of the stimulus train (p = 0.87). Error bars depict SEM.

A distinctive feature of the human condition is that following brief maximal voluntary muscle contraction, the CMAP amplitude is less depressed, typically for several minutes [17, 18]. Since a decrease in release probability could result in less depression, we assessed this unique phenotype in *Drosophila*. We measured the amplitude of evoked release at the larval neuromuscular junction following a 10 Hz stimulation for 2 seconds. Responses were normalized to the initial EJP response to account for the decrease in EJP amplitude seen in the *P[syt^P-L^]* heterozygotes. Beginning at the fourth pulse of this stimulation period, *P[syt^P-L^]* heterozygotes exhibited a significant 5–10% decrease in synaptic depression relative to control (Fig 6, S2 Table). However, when a test stimulation was applied 1 minute after the end of the stimulus train, this effect was no longer observed; the amplitude of the evoked response in both the *P[syt^P-L^]* heterozygotes and the controls had returned to resting levels (Fig 6, S2 Table).

**Fig 7:**
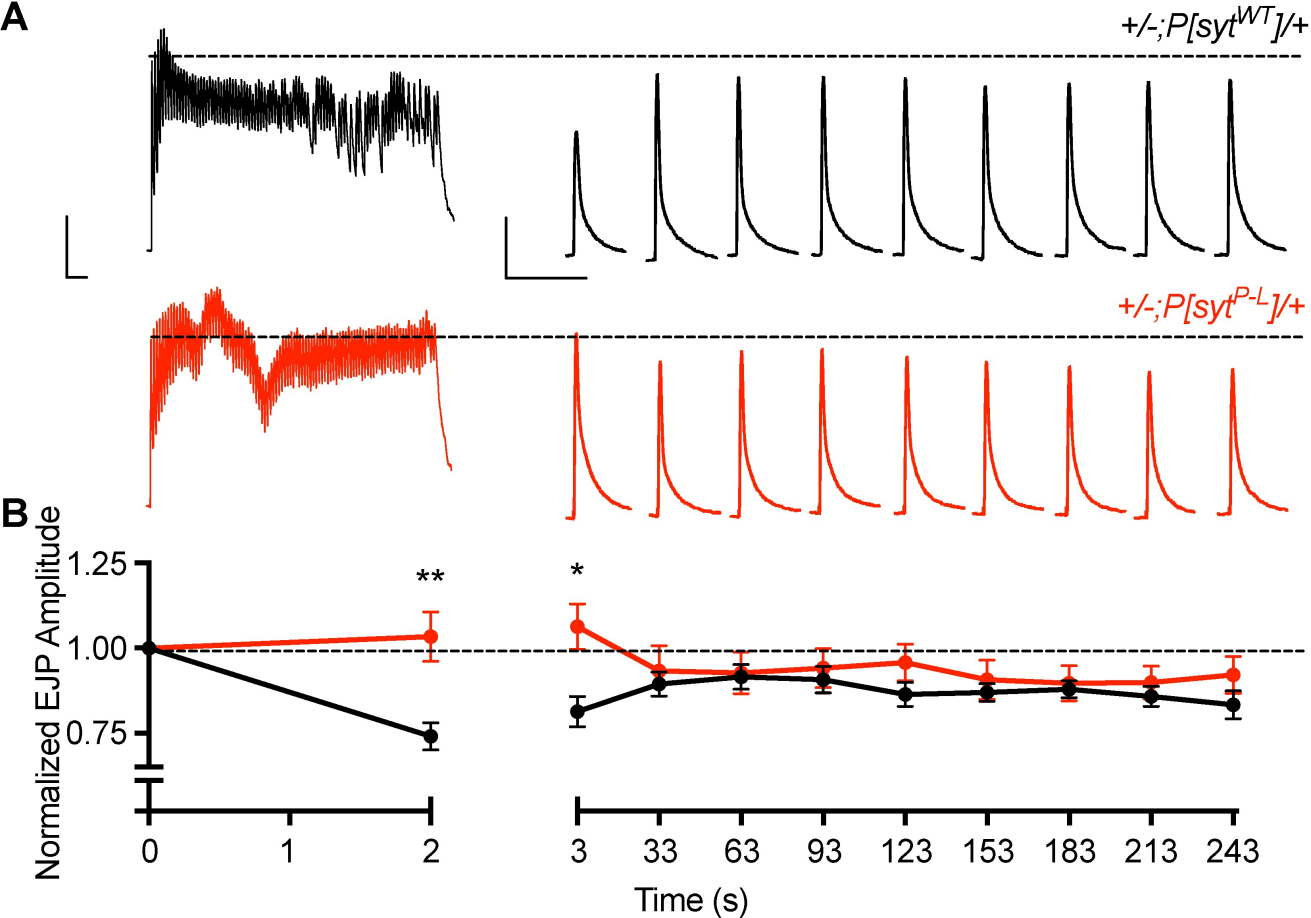
The *syt^P-L^* mutation does not result in synaptic depression during and shortly after a high frequency 50 Hz stimulation, but this relative increase in release is short-lived. (A) Representative traces for control and *P[syt^P-L^]* heterozygotes showing the initial 2 second 50 Hz train, followed by each post-train pulse at 1 second, then every 30 seconds thereafter for 4 minutes. Dotted line represents the amplitude of the initial response. Scale bar represents 10 mV, 0.1 s. (B) Average EJP amplitude during and after 50 Hz stimulation in control (n = 18) and *P[syt^P-L^]* heterozygotes (n = 24). Responses were normalized to the amplitude of the initial response. Unlike controls, *P[syt^P-L^]* heterozygotes do not exhibit synaptic depression at the end of the stimulus train (p << 0.0001) and 1 second after cessation of the train (p = 0.0012). Error bars depict SEM.

Since maximum voluntary contraction can produce motor nerve firing rates in excess of 50 Hz [27], larval preparations were stimulated at 50 Hz for 2 seconds in an attempt to more closely approximate the stimulus that results in the prolonged increase in release seen in the affected family members. Normalized post-stimulus train responses were tested at 1 second and then at 30-second intervals out to 4 minutes. This protocol displayed similar results to the 10 Hz stimulation experiment. Both at the end of the stimulus train and at 1 second post-stimulation, there is no synaptic depression in the *P[syt^P-L^]* heterozygotes, unlike in the control (Fig 7, S3 Table). However, normalized responses to test stimulations at 30 seconds or longer after cessation of the stimulation train showed no significant differences in release between the *P[syt^P-L^]* heterozygotes and controls (Fig 7, S3 Table). Thus, our transgenic model system cannot provide insight into the prolonged increase of the CMAP amplitude following maximal voluntary contraction, which can last 10 minutes or longer in humans [17, 18].

### Basal levels of locomotor activity are decreased in *P[syt^P-L^]* heterozygotes

**Fig 8:**
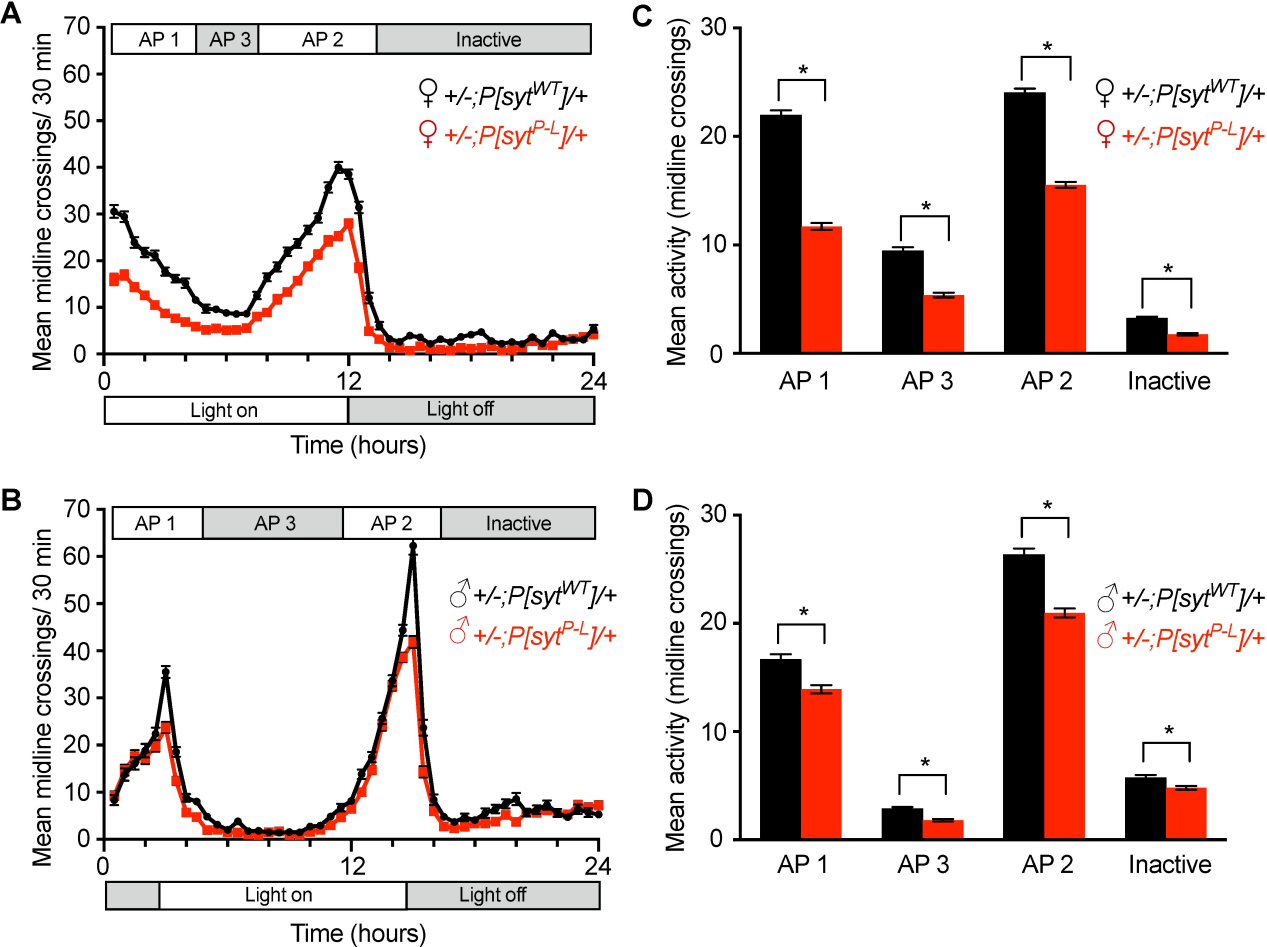
The *syt^P-L^* mutation affects locomotor activity in *Drosophila*. (A, B) Average activity per 30 minutes for an averaged 24-hour day in a 6-day *Drosophila* Activity Monitoring Assay in 5–7 day old females (A) and males (B). (C, D) Distinct activity periods (AP) were averaged for females (C, n = 48 for each genotype) and males (D, n = 48 for *+/-;P[syt^P-L^]/+*, n = 45 for control). *P[syt^P-L^]* heterozygotes were significantly less active than controls during all activity periods in both males and females (* indicates p < 0.001, student t-tests). Error bars depict SEM.

The P-L familial condition presents with distal limb deformities, muscle wasting, and difficulty walking [17, 18]. To assess the impact of this mutation on overall motor function in our model system, the *Drosophila* activity-monitoring (DAM) assay was performed to quantify basal locomotor activity levels. This assay determines total activity over time. However, as activity levels in *Drosophila* males and females are known to have different circadian cycles [28, 29], males and females were tested separately. Activity level was determined by monitoring individual flies for 6 days in a 12hr light: 12hr dark cycle (Fig 8A and 8B) in age- and sex-matched adult *Drosophila P[syt^WT^]* or *P[syt^P-L^]* heterozygotes.

*Drosophila* are typically active during periods of light and inactive during periods of dark. Furthermore, flies have an increase in locomotor activity in anticipation of the transition between dark to light and light to dark. We analyzed the locomotor activity at the two anticipatory peaks of activity (Active 1 and 2, respectively), the non-peak period during lights on (Active 3) and the inactive period approximately corresponding to lights off (Inactive). We find distinct periods of activity between males and females, consistent with published data [28, 29]. During all activity periods, both male and female *P[syt^P-L^]* heterozygotes displayed significantly less movement (Fig 8C and 8D, S4 Table).

### The rate of muscle fatigue is faster in *P[syt^P-L^]* heterozygotes

**Fig 9:**
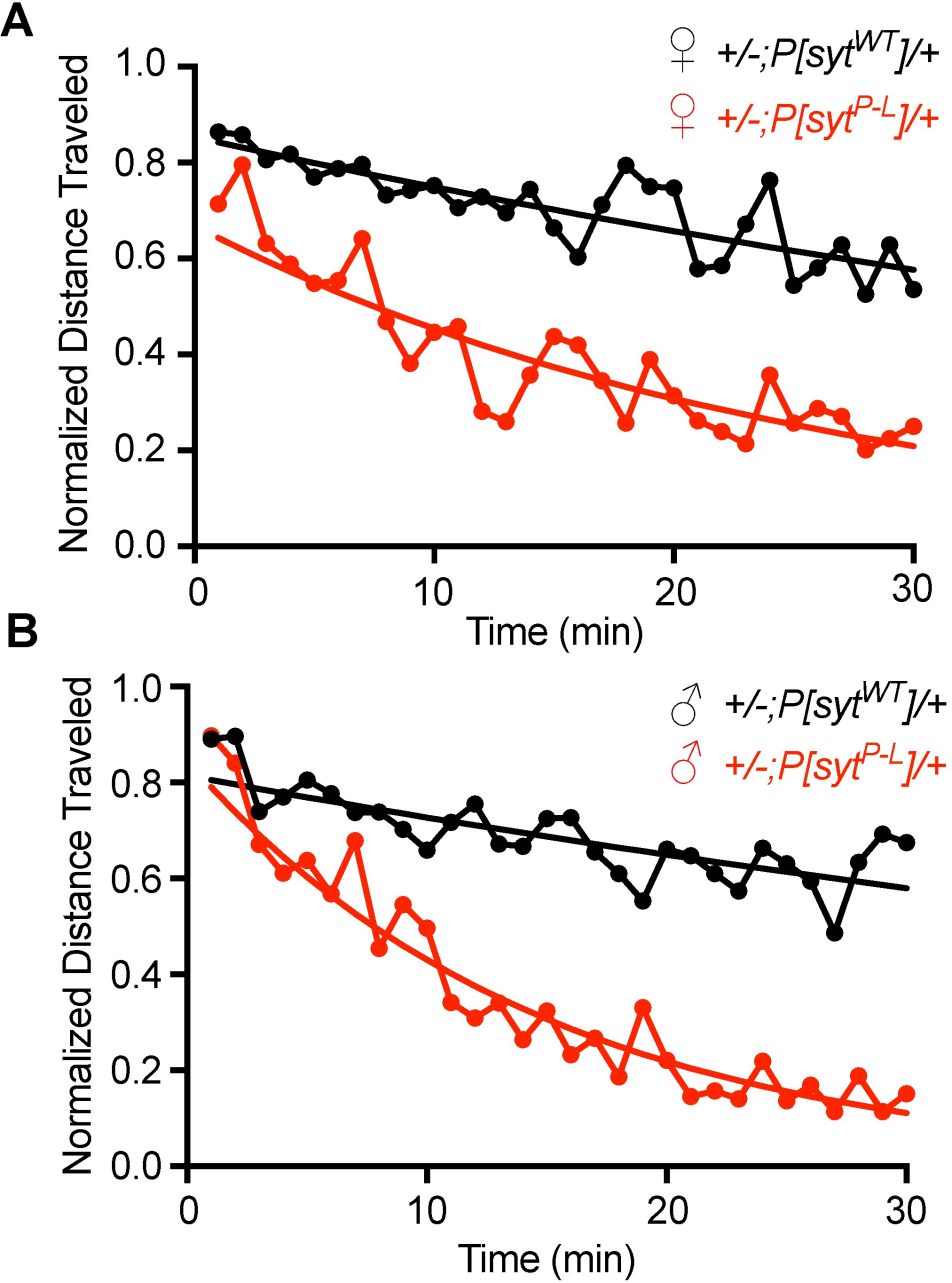
The *syt^P-L^* mutation increases the rate of fatigue in *Drosophila*. (A, B) Normalized values of distance traveled up a vial wall after repetitive drops over time for 5–7 day old females (A) and males (B). Each point is a binned 1 minute average of 6 drops (1 drop every 10 seconds) normalized to the percentage of the distance traveled after the first drop. Statistical analysis indicates different rates of fatigue (see methods) between the two genotypes for both females (p < 0.0001) and males (p < 0.0001), indicating the *P[syt^P-L^]* heterozygotes (n = 10) fatigued significantly faster than controls (n = 10).

Affected individuals with the human disease also present with cramping and pain upon physical exertion, difficulty with sports, and some fatigable eye movements [18]. Since these can all be associated with fatigability in muscle, we analyzed fatigue rate in adult *Drosophila* using a modified climbing assay [30]. Flies were repetitively knocked to the bottom of a vial. This stimulates negative geotaxis, an innate escape response in which flies climb up the vial wall. Vials were dropped (see methods) every 10 seconds for 30 minutes. After each drop, the distance each fly climbed upward within 5 seconds was measured and averaged. As with the DAM assay, we tested age-matched female and male adult flies separately. Differences were observed as early as the first drop for both males and females of *P[syt^P-L^]* heterozygotes and controls (female *+/-;P[syt^WT^]/+* = 6.49 ± 0.60 cm, female *+/-;P[syt^P-L^]/+* = 3.67 ± 0.91 cm, p = 0.017, student t-test, male *+/-;P[syt^WT^]/+* = 6.99 ± 0.45 cm, and male *+/-;P[syt^P-L^]/+* = 4.68 ± 0.64 cm, p = 0.009, student t-test, average distance traveled ± SEM after the first drop). To specifically measure fatigue, we controlled for the decreased overall activity in the mutants by normalizing all measurements to the distance traveled after the initial drop. The normalized data was then graphed over time for both females and males (Fig 9A and 9B, respectively). As a measure of the rate of fatigue, we calculated the log of the raw data and used linear regressions to provide predicted lines of best fit (not shown). The slopes of these lines were statistically significantly different between mutants and controls in both females and males (slopes for female *+/-;P[syt^WT^]/+* = -0.013, female *+/-;P[syt^P-L^]/+* = -0.039, male *+/-;P[syt^WT^]/+* = -0.011, and male *+/-;P[syt^P-L^]/+* = -0.069, p < 0.0001 for both females and males, see methods). These results demonstrate a faster rate of fatigue in the *P[syt^P-L^]* heterozygotes, consistent with the muscle fatigability seen in the affected family members.

## Discussion

In this study we have investigated a synaptotagmin point mutation that was implicated in the etiology of a congenital myasthenic syndrome found in a single family. By modeling this point mutation *in silico*, we predict significant conformation changes to a key, well-ordered Ca^2+^-binding residue essential for fast, synchronous neurotransmitter release. The role of this conserved proline residue in these C_2_ domains could be to punctuate the end of the β-strand leading into the Ca^2+^-binding pocket thereby providing maximum Ca^2+^ binding volume optimizing Ca^2+^ sensitivity.

We expressed the homologous mutation in *Drosophila* and documented several similar deficits to those seen in the affected patients. In addition, we found a decreased Ca^2+^ affinity for release and decreased release probability that are consistent with the conformational changes predicted by our *in silico* analysis. Notably, this *syt^P-L^* mutation not only fails to rescue synaptotagmin function, it has a negative impact on survival when expressed as the sole source of synaptotagmin *(-/-;P[syt^P-L^]/+*); the survival rate is significantly lower than that of larvae with no synaptotagmin expression (*-/-*). This finding demonstrates for the first time that this proline residue in the C_2_B Ca^2+^-binding pocket is essential for synaptotagmin’s Ca^2+^-sensing function. By driving expression of the mutant transgene in *syt* heterozygotes (*+/-;P[syt^P-L^]/+*), we were able to achieve expression levels of *syt^WT^* and *syt^P-L^* similar to that in the human disease.

Electrophysiological analyses demonstrated deficits that may help inform the human condition. Consistent with the human nerve conduction tests, the EJP amplitude at the larval neuromuscular junction in *P[syt^P-L^]* heterozygotes is significantly decreased. This decrease in the initial evoked response is not due to a decrease in quantal size or the readily releasable pool. *P[syt^P-L^]* heterozygotes exhibit a decreased Ca^2+^ affinity, as documented by the increased EC_50_ for Ca^2+^, which resulted in a decreased release probability, as documented by the facilitated responses in paired pulse experiments, and either no, or less, depression during high frequency stimulations. This decreased probability of release could account for an increased failure rate at the neuromuscular junction, consistent with the decreased CMAP seen in humans.

The *P[syt^P-L^]* mutation resulted in significant electrophysiological deficits when expressed in a heterozygous background. This is notable, as synaptotagmin heterozygotes previously have been shown to exhibit no significant changes in electrophysiological responses compared to synaptotagmin homozygotes [31]. Although we did not express this mutation in an otherwise wild type background, this would be consistent with a dominant negative phenotype.

This proline to leucine mutation presents with behavioral deficits similar to those in patients, most notably decreased movement and increased fatigability. This similarity of multiple deficits found in the familial disease strongly supports a role for this residue in the etiology of this form of congenital myasthenic syndrome. It is only recently that mutations in the *syt1* and *syt2* genes have been implicated in human disease [17, 18, 32]. A patient with a *de novo* mutation in the human *syt1* gene presented with an early onset dyskinetic movement disorder, severe motor delay, and profound cognitive deficits attributed to a single amino acid substitution in the C_2_B Ca^2+^-binding pocket: an isoleucine to threonine (I-T) substitution [32]. This hydrophobic isoleucine residue had been shown previously to mediate Ca^2+^-dependent membrane penetration by synaptotagmin [33–36]: an effector interaction critical for coupling Ca^2+^ influx with neurotransmitter release from neurons [37, 38]. Since this *syt* mutation results in the most severe deficits in an animal system [37] and is located in the *syt1* gene, which is preferentially expressed in the cerebral hemispheres [15, 16], it is not surprising that this patient experienced extreme cognitive deficits as well as the most severe motor deficits observed in a synaptotagmin human disease to date [32].

Herrmann et al, 2014 reported two mutations in the human *syt2* gene implicated in the etiology of congenital myasthenic syndrome patients: the proline to leucine (*syt^P-L^*) mutation analyzed in this paper and an adjacent aspartate to alanine (*syt^D-A^*) mutation. Both of these highly conserved residues are located in synaptotagmin’s C_2_B Ca^2+^-binding pocket [9, 19, 39]. While the function of this proline residue had not been previously studied in animal models, previous studies have demonstrated that the Ca^2+^-binding aspartate residues of the C_2_B domain are essential for synaptotagmin function [4, 14, 17, 35], at times resulting in lethality in animal models [4, 17]. The single viable *Drosophila* line from our lab investigating C_2_B aspartate mutations had somewhat reduced expression of the mutant transgene [4]. Since these mutations result in a dominant negative decrease in evoked transmitter release (worse than no synaptotagmin at all) [4], the decreased expression level in our line may have contributed to its viability. When driven in the presence of native synaptotagmin, our *Drosophila* lines that disrupted Ca^2+^ binding by C_2_B were viable, but they did result in a significant dominant negative knockdown in function [4]. For the *syt^D-A^* familial mutation, expression of the mutant syt from a transgene in *syt* heterozygotes resulted in lethality in 4 of 6 independent transgenic lines. There was a dramatic decrease in the evoked response in the remaining two lines [17]. The *syt^P-L^* mutation investigated here, however, resulted in no viability issues in the heterozygous background, and only a 20% decrease in the evoked response. Thus, it is surprising that both the *syt^P-L^* and the *syt^D-A^* human families present with such similar symptoms [17, 18]. Given the severe consequences of mutating the C_2_B Ca^2+^-binding aspartate residues in animal models, it is noteworthy that the deficits seen in the affected human family are comparatively mild [17, 18].

It has been shown that Ca^2+^-binding by the aspartate residues [9, 19, 35] as well as membrane penetration by the hydrophobic residue [33–36], both located in the C_2_B domain of synaptotagmin, are of critical importance for proper synchronous transmitter release [4, 14, 17, 18, 32, 37]. Interestingly, the proline residue investigated in this study is located directly adjacent to one of these aspartates residues. We speculate that this proline residue may provide conformational rigidity important for stabilizing the C_2_B Ca^2+^-binding pocket. By mutating the proline to a leucine, the rigid R group found in the proline residue would be lost, potentially affecting the precise conformation of the pocket and impacting the ability of the adjacent aspartate to bind Ca^2+^. Such a mechanism could result in a decreased, albeit not demolished, ability of the C_2_B domain to bind Ca^2+^, resulting in the less severe phenotype documented here compared to both the aspartate and isoleucine mutations *in vivo* [4, 11, 17, 18, 32, 37].

In our *Drosophila P[syt^P-L^]* heterozygotes, the impact of high frequency stimulation on EJP amplitude was short-lived. Thus our model system cannot provide insight into the long-lasting decrease in depression of the CMAP seen in the human patients following maximal voluntary contraction. The initial facilitation and following lack of depression observed in our *P[syt^P-L^]* heterozygotes during and immediately after high frequency stimulation is predicted by the residual Ca^2+^ hypothesis for short-term plasticity [40–42]. The increase in paired pulse facilitation (Fig 5D) and lack of depression observed during high frequency stimulation (Figs 6 and 7) demonstrate that the *syt^P-L^* mutation decreased release probability compared to wild type. Accordingly, the build-up of Ca^2+^ that occurs during high-frequency stimulation is predicted to contribute to a relatively increased release probability during and shortly after the stimulus train. As this *syt^P-L^* mutation is predicted to disrupt the critical C_2_B Ca^2+^-binding pocket of synaptotagmin (Fig 1A–1C), which is the Ca^2+^ sensor for fast, synchronous neurotransmitter release, a decrease in release probability was not unexpected. The note-worthy long-lasting potentiation of the CMAP, which can last up to 60 minutes in human *syt^P-L^* patients [18] remains unexplained; mutation of the Ca^2+^ sensor would not be predicted to cause such a long-lasting effect.

The decreasing cost and increasing availability of DNA sequencing has led to an increased incidence of genomic sequencing during patient diagnoses. This ever-increasing process will undoubtedly lead to the discovery of additional synaptic mutations and associated genetic disorders. The need for a relatively quick and cost effective method to begin elucidating the molecular mechanisms underlying these synaptic disorders is vital. Using *Drosophila* to model these newly discovered conditions in highly conserved pathways like synaptic transmission is ideal for elucidating their underlying mechanisms, as *Drosophila* provide a cost-effective and rapid mechanism for analysis.

In summary, we have identified the functional role of a predicted leucine-induced conformational change in the C_2_B Ca^2+^-binding pocket of synaptotagmin. Expression of the homologous human mutation in *Drosophila* resulted in deficits similar to many of the human symptoms. Thus, this work supports a role for this synaptotagmin point mutation in disease etiology and elucidates the molecular mechanisms responsible.

## Materials and methods

### Mutagenesis

In *Drosophila*, the *syt1* gene is expressed in all neurons [23, 24] and is homologous to both *syt1* and *syt2* in mammals [21]. Proline residue 363 of *Drosophila syt1* is homologous to proline 308 in human *syt2*, implicated in the human disease, and proline 310 in rat *syt1*, modeled in Fig 1. Using the *Drosophila syt1* coding sequence [43, 44], both a wild type control and a P363L mutant cDNA flanked by EcoRI and BglII restriction sites were synthesized by GeneWiz (La Jolla, CA). The synthesized cDNAs were then subcloned into the PUAST-attB vector to place the transgenes under the control of the UAS promoter and the PhiC31 targeted insertion system was used to target the transgene containing vectors to the attP2 landing site on the *Drosophila* third chromosome [BestGene, Chino Hills, CA; [45, 46]]. We refer to the mutant transgene as *P[syt^P-L^]* and the control transgene as *P[syt^WT^]*.

### Fly lines

Expression of the UAS-driven *syt* transgenes was localized to the nervous system using a pan-neuronal source of GAL4 [*elavGAL4* [47, 48]]. *Syt^AD4^* was the *syt^null^* mutation used [44]. Synaptotagmin is found natively on the second chromosome, and the *syt* transgene used was incorporated into a third chromosome landing site. To directly assess the function of the *syt^P-L^* mutation, the impact of transgene expression on survival was assessed in the *syt^null^* background. For these experiments, the following genotype *yw;syt^null^P[elavGal4w^+^]/CyO,GFPw^+^* was crossed to *yw;syt^null^/CyO,GFP;P[UASsyt^WT^,y^+^w^+^]* or *yw;syt^null^/CyO,GFP;P[UASsyt^P-L^ y^+^w^+^]* or *yw;syt^null^/CyO,GFP* to generate: 1) the transgenic control in the *syt^null^* background *yw;syt^null^P[elavGAL4w^+^]/syt^null^;P[UASsyt^WT^y^+^w^+^]/+* (referred to as *-/-; P[syt^WT^]/+*), 2) the transgenic mutant in the *syt^null^* background, *yw;syt^null^P[elavGAL4w^+^]/syt^null^;P[UASsyt^P-L^y^+^w^+^]/+* (referred to as *-/-;P[syt^P-L^]/+*), and 3) *syt^null^* mutants, *yw; syt^null^P[elavGAL4w^+^]/syt^null^* (referred to as *-/-*), respectively. Expression of the mutant transgene resulted in embryonic or first instar lethality. Therefore, for most experiments, the *P[syt^P-L^]* transgene was expressed in a *syt* heterozygous background in order to mimic the heterozygous nature of the affected human population (*syt^P-L^/syt^WT^*). This results in synaptotagmin expression from a single copy of the endogenous *syt* gene and from a single copy of the mutant *syt* transgene. For these experiments, the following genotype *yw;syt^null^P[elavGal4w^+^]/CyO,GFPw^+^ was crossed to yw;+;P[UASsyt^P-L^y^+^w^+^]* or *yw;+;P[UASsyt^WT^ y^+^w^+^]* (note: both of these transgenic lines are homozygous for *syt^WT^* on the second chromosome) to generate: 4) the transgenic mutant in the *syt* heterozygous background, *yw; syt^null^P[elavGAL4w^+^]/syt^WT^;P[UASsyt^P-L^y^+^w^+^]/+* (labeled as *+/-;P[syt^P-L^]/+* in the figures and referred to as *P[syt^P-L^]* heterozygotes in the text), and 5) the transgenic control in the *syt* heterozygous background, *yw;syt^null^P[elavGAL4w^+^]/syt^WT^;P[UASsyt^WT^y^+^w^+^]/+* (labeled as *+/-;P[syt^WT^]/+* in the figure legends and referred to as *P[syt^WT^]* heterozygotes in the text). For western analyses, the additional genotypes used for comparisons were: 6) *syt* heterozygotes, *yw; syt^null^/syt^WT^; P[UASsyt^P-L^y^+^w^+^]/+* (labeled as *+/-* in Fig 2A), and 7) *syt* homozygotes, *yw; syt^WT^/syt^WT^; P[UASsyt^P-L^y^+^w^+^]/+* (labeled as *+/+* in Fig 2B). The experimental *syt* heterozygotes (*+/-* in Fig 2A) and homozygotes (*+/+* in Fig 2B) both contained the *P[syt^P-L^]* transgene but lack the *elavGal4w^+^* driver, so the transgene was not expressed.

### Molecular dynamics

As the crystal structures of mammalian synaptotagmin 2 proteins have not been solved, we obtained the crystal structures of rat Syt1 C_2_B (PDB file1TJX) [20] from the Protein Data Bank. The C_2_B domains of mammalian synaptotagmin 1 and synaptotagmin 2 are >90% identical (8 substitutions in the core of 93 C_2_B residues) and most of these substitutions are conservative. We generated models of the P-L mutation using the mutagenesis wizard in Pymol Molecular Graphics System, Version 1.8, Schrödinger, LLC. The QuikMD utility in NAMD [49] was used for the simulation. The structures were solvated using the solvate TCL utility in a cube of approximately 7,000 water molecules (TIP3) with a positive and negative padding of 10 Å on each axis. Residue 359 in C_2_B was restrained with harmonic constraints to dampen coordinate drift within the simulation box. Both wild type and mutant C_2_B models were simulated using NAMD [49] with different random seeds for 50,000,000 frames with a time step of two femtoseconds (total of 100 ns) and a write frequency of 25,000. Periodic boundary conditions were used with PME and a grid spacing of 1.0. We used rigid bonds and constant group pressure control. The pressure was controlled with a Langevin piston set at 1.01325 bar (atmospheric pressure). The temperature was controlled at 310 K with Langevin dynamics and a dampening coefficient of 5 ps^-1^ was applied to each trajectory. We validated that the trajectories reached equilibrium by calculating the root mean squared displacements of all atoms in the protein after alignment over the course of the run. Each unique simulation setup was run three times.

### Lethality assay

The only source of synaptotagmin from the native locus (*syt^WT^*) in the crosses used to assess the effect of transgene (*P[syt]*) expression was located on the *CyO,GFP* balancer chromosome (see fly lines section above). Therefore, any progeny that were homozygous for native synaptotagmin are categorized as unhatched since they are also homozygous for *Cy*, which is embryonic lethal (Table 1, S2 Fig). Based on Mendelian genetics, the expected frequency is ~25% (S2 Fig). At least three bottle crosses using at least 50 virgin females each were fed on molasses plates supplemented with a dab of yeast/baby food mixture and were left to mate for two days. A fresh molasses plate dabbed with yeast/baby food mixture was then provided and the flies were allowed to lay eggs for 6 hours to provide age-matched progeny. A total of 4476 eggs were collected and observed. First instar progeny were then scored by phenotype, and the number of unhatched eggs was counted. The plates were observed daily for three consecutive days to control for any developmental delays. A chi-squared test between the three genotypes was applied to reject the test of independence. Additional chi-square tests were applied to test for differences between *-/-;P[syt^WT^]/+* and *-/-;P[syt^P-L^]/+*, as well as between *-/-* and *-/-;P[syt^P-L^]/+*.

### Immunoblotting

Western analysis of larval CNSs was used to determine relative levels of synaptotagmin expression. The CNS was isolated from third instars dissected in Ca^2+^-free HL3.1 containing 70 mM NaCl, 5 mM KCl, 4 mM MgCl_2_, 10mM NaHCO_3_, 5 mM Trehalose, 115 mM sucrose, 5 mM HEPES, pH 7.2 [50]. Samples were sonified for 10 pulses using a Branson Sonifier 450 (VWR Scientific, Westchester, PA) in Laemmli buffer (Bio-Rad, Hercules, CA) containing 5% 2-mercaptoethanol. The indicated genotype was loaded one CNS per lane. Samples were electrophoresed, transferred to Immobilon membranes (Millipore, Bedford, MA), and washed in blocking solution according as described [31]. Blots were probed overnight at 4°C with an anti-synaptotagmin antibody, Dsyt-CL1 [4], diluted 1:2500 and an anti-actin antibody, MAB 1501 (Millipore Bioscience Research Reagents, Billerica, MA), diluted 1:10,000. Actin levels were used to normalize for equal protein loading. Blots were visualized using an EpiChemi^3^ Darkroom and Labworks Imaging software (UVP BioImaging, Upland, CA). The synaptotagmin:actin ratio was determined for each lane, then normalized to the mean synaptotagmin:actin ratio of all control lanes on an individual blot to allow comparison of signal between multiple blots. Outliers in total protein loaded (as indicated by actin levels) were not included in the analysis. Statistical significance for each comparison was determined using a student’s t-test.

### Immunolabeling

For immunolabeling of the neuromuscular junction, third instars of *P[syt^WT^]* heterozygotes and *P[syt^P-L^]* heterozygotes were dissected in Ca^2+^-free HL3.1 saline to expose their body wall muscles and fixed in phosphate-buffered saline (PBS, 137 mM NaCl, 1.5 mM KH_2_PO_4_, 2.7 mM KCl, 8.1 mM Na_2_HPO_4_) containing 2% formaldehyde for 1 hour. Whole-mounts were incubated overnight in Dsyt-CL1 diluted 1:400 in dilution media [PBS with 0.1% Triton, 1% bovine serum albumin, and 1% normal goat serum (NGS from Jackson ImmunoResearch, West Grove, PA)], washed in PBST (PBS with 0.1% Triton) for 3 hours, incubated in Alexa Fluor 488 goat-anti-rabbit antibody (Invitrogen, Carlsbad, CA) diluted 1:400 and Texas Red anti-HRP (Jackson ImmunoResearch, West Grove, PA) diluted 1:50 in dilution media for 1 hour, washed in PBST for 1 hour, and mounted in Citifluor (Ted Pella, Redding, CA). To label transgenic synaptotagmin expressed in the *syt^null^* background, the above protocol was applied to first instars rather than third instars with the following changes: first instars were fixed in PBS containing 4% formaldehyde for 2 hours and Texas Red anti-HRP was omitted. Neuromuscular junctions at the muscle 6/7 junction were imaged for both first and third instars with a Zeiss 880 light scanning microscope using a 40X objective, and acquired using Zeiss Zen 2.1 acquisition software, version 11,0,3,190.

### Longevity assay

Lifespans between *+/-;P[syt^WT^]/+* and *+/-;P[syt^P-L^]/+* were recorded from at least three separate crosses using at least 50 virgins in each cross. Lenth’s analysis was used to determine adequate sample size. N = 151 for *+/-; P[syt^WT^]/+* and n = 149 for *+/-; P[syt^P-L^]/+*. Ten newly hatched adult flies/vial were observed/flipped into fresh food every Monday/Wednesday/Friday and number of living or dead flies were recorded. Flies that were found alive but stuck in the food were omitted from analysis. Any flies that escaped during flipping were also omitted from analysis, as there was no way to determine the length of natural life. The Wilcoxon (two-sample) Rank-Sum test was used to determine significance between genotypes.

### Excitatory junction potentials

Third instars were dissected in Ca^2+^-free HL3.1 saline. EJPs were evoked from muscle fiber 6 of abdominal segments 3 and 4 in HL3.1 containing 1.0 mM Ca^2+^ using standard techniques [25, 31, 37]. Intracellular electrodes of 10–25 MΩ contained 3 parts 2M potassium citrate to 1 part 3M potassium chloride. The resting membrane potential of each fiber was maintained at -55 mV by passing no more than 1 nA of current. Nerves were stimulated with a suction electrode filled with HL3.1 containing 1.0 mM Ca^2+^. All events were collected using an AxoClamp 2B (Molecular Devices, Sunnyvale, CA) amplifier and digitized using a Powerlab 4/35 A/D converter (ADInstruments, Sydney, Australia). EJPs were recorded in LabChart software (ADInstruments, Sydney, Australia). For single evoked responses, averages of 10 EJPs collected at 0.04 Hz were calculated for each individual fiber. Statistical significance was determined using a student’s t-test. Spontaneous events were identified manually after recordings had been randomized and blinded to the researcher. Average mEJP amplitudes were determined from 50 consecutive mEJP events/fiber, taken from consistent time periods across fibers. Statistical significance for mEJP amplitude was determined by the Wilcoxon Rank-Sum test. Frequency of spontaneous events was determined by manually counting number of mEJP events during one minute of baseline activity/fiber. Each fiber was analyzed for the same time period during the recording to eliminate bias.

### Ca^2+^ dependence of release

Individual fibers were bathed in HL3.1 saline containing Ca^2+^ levels varying from 0.05 mM–5.0 mM. Each fiber was recorded in at least three different Ca^2+^ concentrations to be included in analysis. At each Ca^2+^ concentration, the mean response of 5 EJPs collected at 0.5 Hz were calculated. Lines of best fit were calculated using a nonlinear regression analysis, which provided EC_50_ values and their confidence intervals for each genotype.

### Paired pulse

Experiments were conducted using a 0.02 second delay between stimulations, and averages of EJP responses were normalized to the first stimulation. Statistical significance was determined using a student t-test.

### High frequency stimulation

Assays to observe depression or facilitation were first assessed by stimulating the nerve at 10 Hz for 2 seconds followed by a test pulse 60 sec post-stimulus train. EJP amplitudes were normalized to that of the first EJP prior to calculating the average response within a genotype. A repeated measures analysis of variance was used to compare the two genotypes at each stimulus. Post-hoc t-tests were then used to determine significance. To more closely mimic maximal voluntary contraction at the human neuromuscular junction, another protocol was designed using 50 Hz stimulation for 2 seconds, followed by test pulses at 1 second post-stimulus train, and then at 30 second intervals to 4 minutes. Similar statistical methods as above were applied to determine significant differences between amplitudes at each individual time point.

### Hypertonic solution stimulations

A PicoSpritzer III was used to administer a puff application of either 0.5M sucrose or HL3.1 bath solution to the junctional region of muscle fibers 6 at 5 pounds per square inch (psi) for 10 seconds. Hypertonic stimulations were conducted in Ca^2+^-free HL3.1. mEJP events were counted manually for all time points analyzed. To determine baseline mEJP frequency, 10 seconds of mEJP events immediately prior to sucrose or HL3.1 application were analyzed. To determine average mEJP frequency during maximum sucrose stimulation, 15 seconds of mEJP events were analyzed, beginning 5 seconds into the sucrose or HL3.1 application. Statistical significance was determined by student t-tests.

For all electrophysiological experiments, post-hoc Lenth’s analyses were performed to ensure adequate sample size. If fibers were unable to maintain a physiological potential when injected with a maximum of 1 nA of current, they were excluded.

### Drosophila activity monitoring

The basal locomotor activity of adult *Drosophila* was assessed using the TriKinetics Locomotor Activity Monitoring System (Trikinetics, Waltham, MA). Age-matched 5–7 day old adult flies of the indicated genotypes were loaded into DAM2 *Drosophila* activity monitors. Males and females were tested separately to control for sex differences in circadian rhythms [28, 29]. Animals were placed on a 12hr light: 12hr dark cycle on standard fly food media and activity was recorded for 7 days using DAM system Acquisition Software. Data from the first day were not included in the analysis to allow flies to acclimate to their new environment. Activity levels were binned into 30-minute intervals and uploaded into Microsoft Excel using DAM Filescan software. Using these 30-minute intervals, an averaged 24-hour day was created for each genotype and sex. Four distinct activity periods were determined post-hoc that correspond to *Drosophila*’s two anticipatory peaks (Active 1 and 2), a lights-on active period (Active 3), and a lights off-inactive period (Inactive). T-tests were performed to compare genotypes during each activity period for both males and females. Flies that died during the course of the experiment were excluded from the analysis. Post-hoc Lenth’s analysis was performed to ensure adequate sample size.

### Fatigability assay

To assess fatigue, a modified negative geotaxis assay was used [30]. For each genotype, 10 un-anesthetized, sex- and age-matched 5–7 day old adult flies were loaded by aspiration into a 9 cm tall standard *Drosophila* vial. Flies were not exposed to CO_2_ to avoid possible side effects and were left to acclimate for 1 hour. Experimental and control vials were secured side by side in a vial rack, raised 6–8 inches above a table, and then dropped, causing all flies to fall to the bottom of the vial. Vials were dropped every 10 seconds for 30 minutes, and a picture was recorded 5 seconds after every drop. Each picture was analyzed in ImageJ for the distance each fly had traveled up the side of the vial. The average for each sex and genotype was calculated for each picture, and the data was binned into 1 minute averages. To control for any genotype-dependent decrease in mobility and thereby specifically assess fatigue, all average distances were normalized to the average distance traveled after the first drop. Linear regressions of the log of the raw data provided predicted lines of best fit for each sex and genotype. A significant interaction between time and genotype (for both sexes) in the log of the raw data indicates that there is a difference between the slopes for the two genotypes. Fitted curves, after back transformation, are shown in Fig 9. Post-hoc Lenth’s analysis was performed to ensure adequate sample size.

## Conclusions

In this study, we investigate the functional role of a synaptotagmin 2 residue implicated in disease. Recently, a neuromuscular disorder has been reported in which a highly conserved proline residue is mutated to a leucine within synaptotagmin. The functional importance of this residue was unknown. Here we model the impact of this mutation on the structure of synaptotagmin and examine the effects of a homologous mutation *in vivo* using *Drosophila*. We found that this mutation is predicted to produce a conformational change in the C_2_B domain of synaptotagmin that would decrease Ca^2+^ binding. When expressed in flies containing no synaptotagmin, this mutation was lethal, demonstrating the critical nature of the examined residue. When expressed in combination with wild type synaptotagmin, we demonstrated a decreased Ca^2+^ affinity for neurotransmitter release and a decreased release probability. These deficits can account for the defects in neuromuscular function and behavior, which are similar to those of the human patients. Thus, these results support a role for this amino acid substitution in human disease etiology.

## Supporting information

S1 Fig Molecular modeling predicts conformational changes in the C_2_B Ca^2+^-binding domain of synaptotagmin. (A) Dynamic Ramachandran Plot of the residues surrounding the P-L mutation in *syt* C_2_B. The top panels are taken from the trajectories from the P-L simulations. The bottom panels are taken from the wild type *syt1* C_2_B simulations. Each dot in each plot represents a time progression from 0–100 ns. Zero ns being the darkest spot and 100 ns being the bluest. Standard Ramachandran boundaries for alpha and beta secondary structure are provided. (B) Histogram showing the range and variation of phi angles of the D2 aspartate residue in wild type and the P-L mutation throughout the 100 ns molecular dynamics trajectories.
(TIFF)

S2 Fig Example cross used for lethality assay in *syt^null^* background. Potential F1 progeny are shown with the expected Mendelian distribution of each genotype, and identification of null, heterozygotes, and homozygotes at the native locus. Due to the use of the CyO balancer, ~25% of F1 progeny in control crosses (*syt* homozygotes at the native locus) are expected to remain unhatched, as they are also homozygous for Cy, which is embryonic lethal. The remaining progeny should present as ~25% lacking GFP (*sytnull* at the native locus) and ~50% GFP (*syt* heterozygotes at the native locus).
(TIFF)

S1 Table *P[syt^P-L^]* heterozygotes exhibit decreased EJP amplitudes at most Ca^2+^ levels. Table providing p-values for each Ca^2+^ concentration tested in Fig 5. *depicts statistical significance.
(DOCX)

S2 Table *P[syt^P-L^]* heterozygotes exhibit less synaptic depression relative to the control throughout a 10 Hz stimulation, but fail to maintain this relative increase in release upon cessation of the stimulus train. Table providing normalized mean responses, SEM, and p-values for all points tested during and after a 10 Hz stimulation train (Fig 6), where *p < 0.05, and **p < 0.001.
(DOCX)

S3 Table *P[syt^P-L^]* heterozygotes do not exhibit synaptic depression during and shortly after 50 Hz stimulation, but this increase in release relative to controls is not prolonged. Table providing normalized mean responses, SEM, and p-values during and after a 50 Hz stimulation train (Fig 7), where **p << 0.0001, and *p < 0.01.
(DOCX)

S4 Table *P[syt^P-L^]* heterozygotes display decreased motor output compared to controls. Table providing mean sensor crossings and SEM acquired during the *Drosophila* Activity Monitoring assay (Fig 8).
(DOCX)
