# Supplementary figures and images for: Drosophila studies support a role for a presynaptic synaptotagmin mutation in a human congenital myasthenic syndrome
Source: PLoS One. 2017 Sep 27;12(9):e0184817. doi: 10.1371/journal.pone.0184817 (PMC5617158; doi:10.1371/journal.pone.0184817)

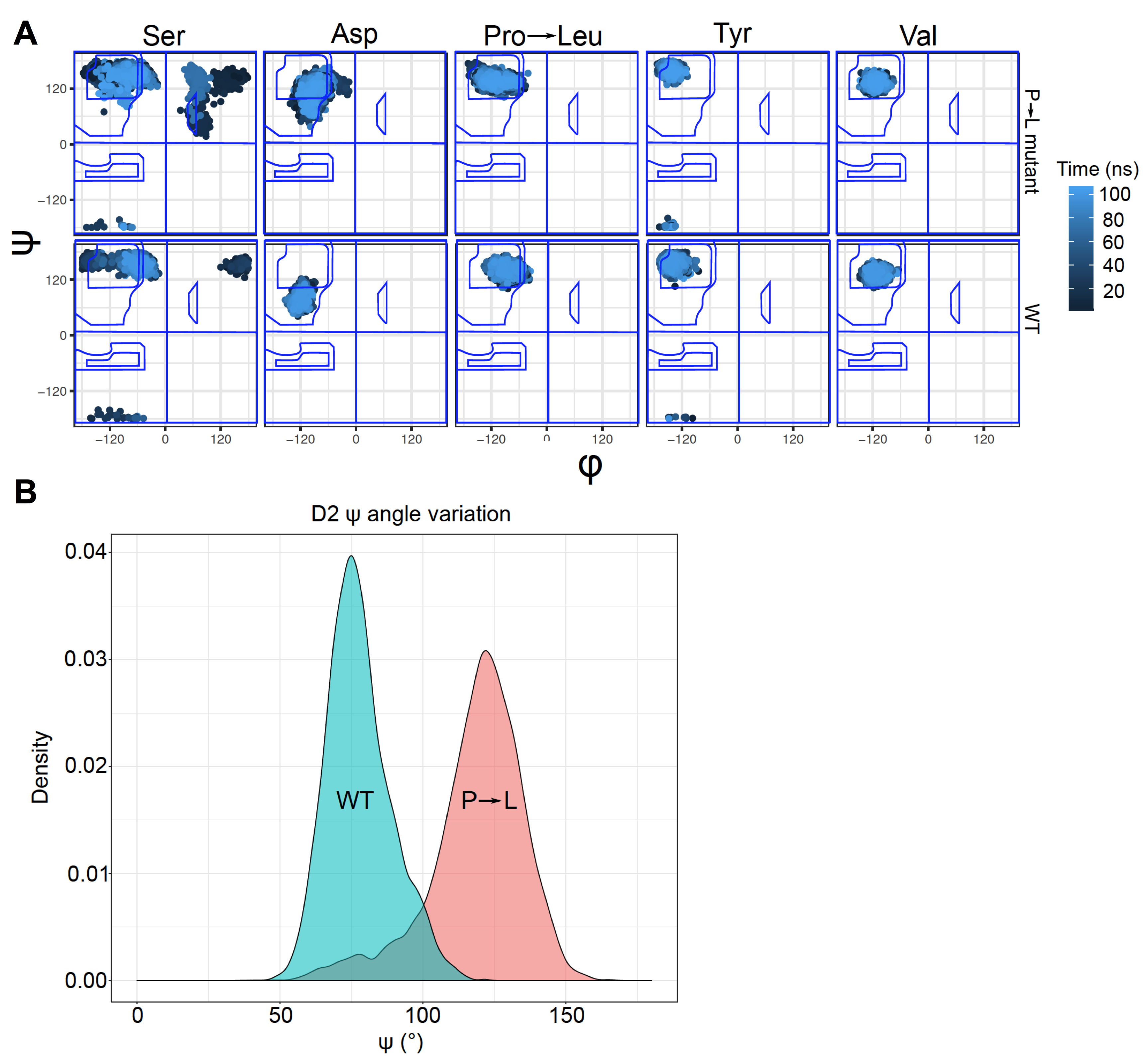

Supplement: S1 Fig — (A) Dynamic Ramachandran Plot of the residues surrounding the P-L mutation in syt C2B. The top panels are taken from the trajectories from the P-L simulations. The bottom panels are taken from the wild type syt1 C2B simulations. Each dot in each plot represents a time progression from 0–100 ns. Zero ns being the darkest spot and 100 ns being the bluest. Standard Ramachandran boundaries for alpha and beta secondary structure are provided. (B) Histogram showing the range and variation of phi angles of the D2 aspartate residue in wild type and the P-L mutation throughout the 100 ns molecular dynamics trajectories. (TIFF) [file pone.0184817.s001.tiff]

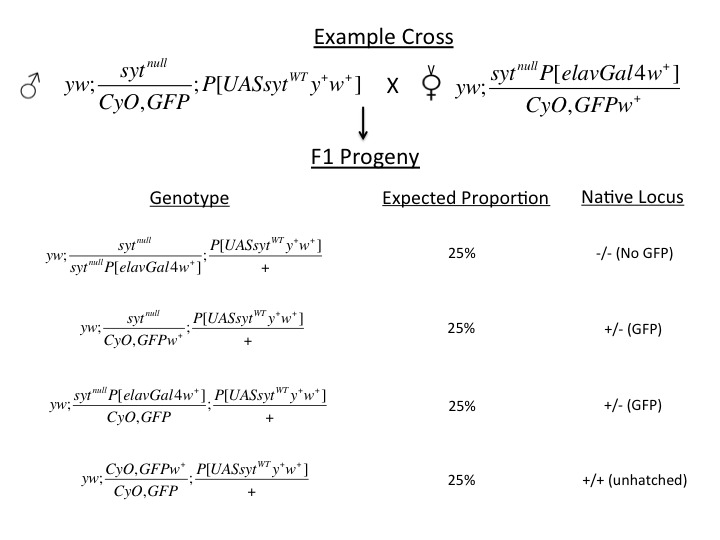

Supplement: S2 Fig — Potential F1 progeny are shown with the expected Mendelian distribution of each genotype, and identification of null, heterozygotes, and homozygotes at the native locus. Due to the use of the CyO balancer, ~25% of F1 progeny in control crosses (syt homozygotes at the native locus) are expected to remain unhatched, as they are also homozygous for Cy, which is embryonic lethal. The remaining progeny should present as ~25% lacking GFP (sytnull at the native locus) and ~50% GFP (syt heterozygotes at the native locus). (TIFF) [file pone.0184817.s002.tiff]
