# Supplementary material for: Drosophila studies support a role for a presynaptic synaptotagmin mutation in a human congenital myasthenic syndrome
Source: PLoS One. 2017 Sep 27;12(9):e0184817. doi: 10.1371/journal.pone.0184817 (PMC5617158; doi:10.1371/journal.pone.0184817)
Supplement: S1 Table — Table providing p-values for each Ca2+ concentration tested in Fig 5. *depicts statistical significance. (DOCX) [file pone.0184817.s003.docx]

|  | *+/-;P[sytWT]/+* | | | *+/-;P[sytP-L]/+* | | |  |
| --- | --- | --- | --- | --- | --- | --- | --- |
| [Ca2+] (mM) | Mean EJP (mV) | SEM | n = | Mean EJP (mV) | SEM | n = | p *=* |
| 0.05 | 0.32 | 0.04 | 12 | 0.28 | 0.06 | 12 | 0.60 |
| 0.1 | 2.86 | 0.95 | 12 | 1.81 | 0.63 | 12 | 0.37 |
| 0.25 | 15.70 | 1.53 | 16 | 10.10 | 1.74 | 16 | 0.02* |
| 0.5 | 27.17 | 0.98 | 23 | 18.16 | 1.60 | 22 | <0.0001* |
| 0.75 | 31.09 | 0.95 | 12 | 23.88 | 2.12 | 10 | 0.003* |
| 1.0 | 35.52 | 0.50 | 15 | 28.40 | 1.08 | 16 | <0.0001* |
| 2.5 | 37.05 | 0.81 | 11 | 31.99 | 2.43 | 11 | 0.05* |
| 5.0 | 39.63 | 0.69 | 13 | 33.32 | 2.51 | 12 | 0.02* |

S1 Table. *P[sytP-L]* heterozygotes exhibit decreased EJP amplitudes at most Ca2+ levels. Table providing p-values for each Ca2+ concentration tested in Fig 5. *depicts statistical significance.
