# Supplementary material for: Drosophila studies support a role for a presynaptic synaptotagmin mutation in a human congenital myasthenic syndrome
Source: PLoS One. 2017 Sep 27;12(9):e0184817. doi: 10.1371/journal.pone.0184817 (PMC5617158; doi:10.1371/journal.pone.0184817)
Supplement: S2 Table — Table providing normalized mean responses, SEM, and p-values for all points tested during and after a 10 Hz stimulation train (Fig 6), where *p < 0.05, and **p < 0.001. (DOCX) [file pone.0184817.s004.docx]

|  | *+/-;P[sytWT]/+* | | *+/-;P[sytP-L]/+* | |  |
| --- | --- | --- | --- | --- | --- |
| Time (s) | Normalized Mean | SEM | Normalized Mean | SEM | p-value |
| 0.1 | 1.00 | 0.00 | 1.00 | 0.00 | 1.0 |
| 0.2 | 0.97 | 0.01 | 1.01 | 0.02 | 0.28 |
| 0.3 | 0.95 | 0.01 | 1.00 | 0.01 | 0.13 |
| 0.4 | 0.93 | 0.01 | 0.99 | 0.02 | 0.033* |
| 0.5 | 0.92 | 0.01 | 1.01 | 0.03 | 0.0013* |
| 0.6 | 0.91 | 0.01 | 0.95 | 0.02 | 0.10 |
| 0.7 | 0.90 | 0.01 | 0.98 | 0.02 | 0.0030* |
| 0.8 | 0.89 | 0.01 | 0.96 | 0.03 | 0.0071* |
| 0.9 | 0.88 | 0.02 | 0.97 | 0.02 | 0.0004** |
| 1.0 | 0.87 | 0.02 | 0.94 | 0.03 | 0.0083* |
| 1.1 | 0.87 | 0.01 | 0.96 | 0.02 | 0.0010* |
| 1.2 | 0.88 | 0.01 | 0.97 | 0.03 | 0.0007** |
| 1.3 | 0.87 | 0.01 | 0.96 | 0.03 | 0.0008** |
| 1.4 | 0.87 | 0.01 | 0.95 | 0.02 | 0.0046* |
| 1.5 | 0.87 | 0.01 | 0.95 | 0.03 | 0.0029* |
| 1.6 | 0.87 | 0.01 | 0.96 | 0.03 | 0.0005** |
| 1.7 | 0.88 | 0.01 | 0.96 | 0.03 | 0.0012* |
| 1.8 | 0.88 | 0.01 | 0.95 | 0.03 | 0.0037* |
| 1.9 | 0.88 | 0.01 | 0.95 | 0.03 | 0.0063* |
| 2.0 | 0.87 | 0.01 | 0.96 | 0.03 | 0.0005** |
| 62.0 | 0.99 | 0.01 | 1.00 | 0.03 | 0.87 |

S2 Table. *P[sytP-L]* heterozygotes exhibit less synaptic depression relative to the control throughout a 10 Hz stimulation, but fail to maintain this relative increase in release upon cessation of the stimulus train. Table providing normalized mean responses, SEM, and p-values for all points tested during and after a 10 Hz stimulation train (Fig 6), where *p < 0.05, and **p < 0.001.
