# Supplementary material for: Drosophila studies support a role for a presynaptic synaptotagmin mutation in a human congenital myasthenic syndrome
Source: PLoS One. 2017 Sep 27;12(9):e0184817. doi: 10.1371/journal.pone.0184817 (PMC5617158; doi:10.1371/journal.pone.0184817)
Supplement: S3 Table — Table providing normalized mean responses, SEM, and p-values during and after a 50 Hz stimulation train (Fig 7), where **p << 0.0001, and *p < 0.01. (DOCX) [file pone.0184817.s005.docx]

|  | *+/-;P[sytWT]/+* | | *+/-;P[sytP-L]/+* | |  |
| --- | --- | --- | --- | --- | --- |
| Time (s) | Normalized Mean | SEM | Normalized Mean | SEM | p-value |
| 0 | 1.00 | 0.00 | 1.00 | 0.00 | 1.00 |
| 2 | 0.74 | 0.04 | 1.03 | 0.07 | <<0.0001** |
| 3 | 0.81 | 0.05 | 1.06 | 0.07 | 0.0012* |
| 33 | 0.89 | 0.04 | 0.93 | 0.07 | 0.50 |
| 63 | 0.91 | 0.04 | 0.93 | 0.06 | 0.54 |
| 93 | 0.91 | 0.04 | 0.94 | 0.06 | 0.62 |
| 123 | 0.86 | 0.04 | 0.96 | 0.05 | 0.19 |
| 153 | 0.87 | 0.03 | 0.91 | 0.06 | 0.60 |
| 183 | 0.88 | 0.02 | 0.90 | 0.05 | 0.73 |
| 213 | 0.86 | 0.03 | 0.90 | 0.05 | 0.52 |
| 243 | 0.83 | 0.04 | 0.92 | 0.05 | 0.21 |

S3 Table. *P[sytP-L]* heterozygotes do not exhibit synaptic depression during and shortly after 50 Hz stimulation, but this increase in release relative to controls is not prolonged. Table providing normalized mean responses, SEM, and p-values during and after a 50 Hz stimulation train (Fig 7), where **p << 0.0001, and *p < 0.01.
