# Supplementary material for: Drosophila studies support a role for a presynaptic synaptotagmin mutation in a human congenital myasthenic syndrome
Source: PLoS One. 2017 Sep 27;12(9):e0184817. doi: 10.1371/journal.pone.0184817 (PMC5617158; doi:10.1371/journal.pone.0184817)
Supplement: S4 Table — Table providing mean sensor crossings and SEM acquired during the Drosophila Activity Monitoring assay (Fig 8). (DOCX) [file pone.0184817.s006.docx]

|  |  | *+/-;P[sytWT]/+* | | *+/-;P[sytP-L]/+* | |
| --- | --- | --- | --- | --- | --- |
|  | Sex | Mean crossings  /30 min | SEM | Mean crossings  /30 min | SEM |
| Active Period 1 | F  M | 21.97  16.70 | 0.43  0.45 | 11.71  13.91 | 0.31  0.38 |
| Active Period 3 | F  M | 9.48  2.89 | 0.32  0.13 | 5.38  1.80 | 0.22  0.11 |
| Active Period 2 | F  M | 24.06  26.38 | 0.35  0.55 | 15.54  20.96 | 0.27  0.42 |
| Inactive Period | F  M | 3.28  5.77 | 0.12  0.23 | 1.77  4.8 | 0.10  0.18 |

S4 Table. *P[sytP-L]* heterozygotes display decreased motor output compared to controls. Table providing mean sensor crossings and SEM acquired during the Drosophila Activity Monitoring assay (Fig 8).
